# Androgen receptor regulates ASS1P3/miR-34a-5p/ASS1 signaling to promote renal cell carcinoma cell growth

**DOI:** 10.1038/s41419-019-1330-x

**Published:** 2019-04-18

**Authors:** Kefeng Wang, Yin Sun, Changcheng Guo, Tao Liu, Xiang Fei, Chawnshang Chang

**Affiliations:** 1 Department of Urology, Shengjing Hospital, China Medical University, 110004 Shenyang, China; 2 George Whipple Lab for Cancer Research, Departments of Pathology, Urology, Radiation Oncology and The Wilmot Cancer Institute, University of Rochester Medical Center, Rochester, NY 14642 USA; 3 Sex Hormone Research Center, China Medical University/Hospital, 404 Taichung, Taiwan

**Keywords:** miRNAs, Long non-coding RNAs

## Abstract

Recent studies have demonstrated that the androgen receptor (AR) could play important roles to promote renal cell carcinoma (RCC) cell proliferation, and other studies have also indicated that suppressing the argininosuccinate synthase 1 (ASS1) could promote proliferation of various tumors. The potential of AR promoting cell proliferation in RCC via altering ASS1, however, remains unclear. Here we found that the expression of ASS1 was lower in RCC tissues than in adjacent normal renal tissues, and a lower ASS1 expression was linked to a worse prognosis in RCC patients. Mechanism dissection showed that AR could decrease ASS1 expression to promote RCC cell proliferation via ASS1P3, a pseudogene of ASS1. The results of RIP assay and AGO2 assay revealed that AR could bind ASS1P3 to increase RCC cell proliferation via altering miR-34a-5p function, which could bind to the 3′UTR of ASS1 to suppress its protein expression. ASS1P3 could function as a miRNA decoy for miR-34a-5p to regulate ASS1 in RCC. Preclinical study also supports the in vitro data. Together, these results demonstrated that ASS1P3 could function as a competing endogenous RNA to suppress RCC cell progression, and targeting this newly identified AR-mediated ASS1P3/miR-34a-5p/ASS1 signaling might help in blocking proliferation.

## Introduction

Siegel et al. reported that renal cell carcinoma (RCC) would account for ~3.77% of new adult malignancies in the United States in 2018^1^. Of all urological tumors, the incidence of RCC is 45.13% and it has a high death rate due to its rapid progression and late diagnosis^1^. Extensive studies indicate RCC like all other human tumors is the result of misregulation at multiple levels, including noncoding RNAs (ncRNAs), epigenetic regulation, or post-translational modifications. However, the detailed molecular mechanisms of the onset and progression of RCC remain largely unclear.

Epidemiology studies indicate there is a gender difference in the incidence of RCC with a male:female ratio of 1.88:1.0^1^, suggesting that sex hormones and/or their receptors may play important roles in the development of RCC. In particular, the androgen receptor (AR) has been shown to be involved in the initiation and development of RCC^2,3^.

Increasing evidence demonstrates that the metabolism of cancer cells helps them adapt to their pathological needs to fuel their accelerated proliferation as well as resistance to apoptosis. Arginine is an important amino acid especially for the young, which plays key roles in many metabolic pathways, such as the production of urea, nitric oxide, and proline^4,5^. Arginine is a nonessential amino acid, which can be converted from citrulline by argininosuccinate synthase 1 (ASS1). However, a loss of ASS1 expression has been found in many tumors, promoting cell proliferation by facilitating activation of pyrimidine synthesis via the CAD (carbamoyl-phosphate synthase 2, aspartate transcarbamylase, and dihydroorotase) complex^6,7^.

The ncRNAs comprise almost 90% of the human transcriptome without protein-coding functions. Recently, many ncRNAs have been identified to be involved in tumor initiation and progression. The microRNAs (miRNAs) are a class of ncRNAs that can bind to the 3′ untranslated region (3′UTR) of the target gene transcripts to suppress the translation or decrease the stability of mRNA^8,9^. Pseudogenes represent another kind of ncRNAs, which constitute a substantial proportion of the “transcriptome”^10^. Their transcription shows tissue-specific features and can be abnormally activated in cancers^11,12^, suggesting that pseudogenes may contribute to tumorigenesis, although the exact mechanisms remain unclear. Recently, Poliseno et al.^13^ have shown that pseudogenes can act as competing endogenous RNAs (ceRNAs) to modulate other RNA transcripts by competing with miRNAs in tumors.

Here we reported that ASS1 and its pseudogene ASS1P3 were abnormally expressed in the clinical RCC samples. Moreover, ASS1P3 could function as a ceRNA facilitated by AR to suppress proliferation by competing with miR-34a-5p in RCC.

## Materials and methods

### Clinical tissues

Clinical human RCC samples were obtained from the Department of Urology, Shengjing Hospital of China Medical University, Shenyang, China. All samples were collected for research purposes. The scientific ethics consent forms were signed by the patients before the study.

### Reagents and materials

GAPDH and AR antibodies were purchased from Santa Cruz Biotechnology (Dallas, TX). ASS1 antibody was purchased from Proteintech Group Inc (Rosemont, IL). Anti-mouse/rabbit second antibodies for western blot were from Invitrogen (Grand Island, NY). Normal rabbit IgG was also from Santa Cruz Biotechnology.

### In vitro cell culture

The OSRC-2, A498, SW-839, Caki-1, and HEK-293 cells were purchased from American Type Culture Collection (ATCC, Manassas, VA). All the cell lines were cultured in Dulbecco’s Modified Eagle’s media (Invitrogen), supplemented with 1% l-glutamine, 10% fetal bovine serum, streptomycin (25 g/ml), and penicillin (25 units/ml); they were all cultured in a 5% (v/v) CO_2_ humidified incubator at 37 °C, and had been tested and authenticated as mycoplasma and bacteria free following ATCC’s instructions during the 3 months before the experiments.

### Lentivirus packaging

The pWPI/pWPI-AR/pLKO.1/pLKO.1-shAR/pLVTHM/pLVTHM-shASS1P3/pLVTHM-miR-34a-5p/pWPI-ASS1P3 plasmids, pMD2G envelope plasmid, and psPAX2 packaging plasmid were transfected into HEK-293 cells using the standard calcium chloride transfection method. The lentivirus soups were collected after incubating for 48 or 72 h and used immediately or frozen in −80 °C for later use.

### RNA extraction and quantitative real-time PCR analysis

Total RNAs were isolated using Trizol reagent (Invitrogen) according to the manufacturer’s instructions and 2 µg RNA used for reverse transcription using Superscript III transcriptase (Invitrogen). Quantitative real-time PCR (qRT-PCR) was applied using a Bio-Rad CFX96 system with SYBR green to determine the mRNA expression level of a gene of interest. The qRT-PCR protocols were as follows: 50 °C for 2 min, 95 °C for 8 min 30 s, followed by 45 cycles at 95 °C for 15 s, and 60 °C for 1 min. The extension was 95 °C for 1 min, 55 °C for 1 min, and 55 °C for 10 s. GAPDH was used as a normalized control.

The miRNAs were extracted using PureLink^®^ miRNA kit and 2 µg RNA used for poly A polymerase at 37 °C for 20 min, and then reverse transcriptase conducted by annealing at 65 °C for 5 min, and at 4 °C for 2 min after adding 50 µm RT anchor primer. The last step was cDNA synthesis at 42 °C for 60 min by adding 2 μl of 10 mM dNTP, 2 μl of 5x RT buffer, 1 μl reverse transcriptase, and ddH_2_O to a total of 20 μl. The qRT-PCR protocol was as follows: 95 °C for 2 min, followed by 45 cycles at 95 °C for 15 s, and 60 °C for 45 s. U6 and/or RPL32 were used as a normalized control.

### Cell proliferation assay

RCC cells were seeded in 24-well plates (3000 cells/well) and cultured at days 2, 4, and 6. The total cell number was calculated using MTT agent. DMSO was used as a control.

### Colony formation assay

Cells were seeded in six-well plates at 48 h after RNA transfection. After 2 weeks, the cells were washed twice with PBS, fixed with 4% paraformaldehyde for 30 min, and then stained with crystal violet for 30 min for visualization and counting.

### Luciferase reporter assay

ASS1 3′UTR involving wild-type or mutant miRNA-responsive elements were cloned into psiCHECK2 vector construct (Promega, Madison, WI) downstream of the Renilla luciferase ORF. SW-839 and OSRC-2 cells were plated in 24-well plates and the cDNAs were transfected with Lipofectamine 3000 transfection reagent (Invitrogen) according to the manufacturer’s instruction. Luciferase activities were measured 36–48 h after transfection by Dual-Luciferase Assay (Promega) according to the manufacturer’s manual.

### Western blot analysis

RCC cells were lysed in lysis buffer on ice, and proteins (50–100 µg) were separated on 10% SDS/PAGE gel and then transferred onto PVDF membranes (Millipore, Billerica, MA). The membranes were blocked by 5% Bovine Serum Albumin (Sigma-Aldrich, St. Louis, MO) for more than 1 h at room temperature and then incubated with proper dilutions of primary antibodies overnight at 4 °C. The next day, the corresponding secondary antibodies were used for 1 h at a concentration of 1:5000 at room temperature and rinsed for 20 min by TBST three times. The bands were visualized by an enhanced chemiluminescence chemiluminescent detection system (Thermo Fisher Scientific, Rochester, NY).

### RNA-pull-down assay

RCC cells were collected with 1.5 ml cell lysis buffer and the qRT-PCR used to detect the concentration of GAPDH to ensure that the lysate input of each group was equal for the next step. Equal amounts of lysates from each group were mixed with 1.5 μl RNase inhibitor and 500 pM antisense oligos overnight at 4 °C before adding 10 μl Streptavidin Agarose beads followed by another rotation for 2 h at 4 °C. The mixture was centrifuged at 3000 rpm for 2 min, and then the beads were washed using cell lysis buffer five times. Total RNAs were extracted by Trizol reagent (Invitrogen) according to the manufacturer’s protocol and subjected to qRT-PCR analysis.

### AGO2 immunoprecipitation

The transfected cells were lysed with RIPA lysis buffer (20 nM Tris-HCl/pH 7.5, 1 mM Na_2_EDTA, 150 mM NaCl, 1% NP-40, 1 mM EGTA, 1% sodium deoxycholate, 1 mM beta-glycerophosphate, 2.5 mM sodium pyrophosphate, 1 mg/ml leupeptin, and 1 mM Na_3_VO_4_) for 30 min on ice. The cell suspension was centrifuged at 14,000 rpm for 15 min. For input, 1/10 supernatant was extracted to detect the protein to ensure that the input of each group was equal for the next step. The remaining 9/10 supernatant was rotated overnight at 4 °C after adding 10 μl beads and 2 μl AGO2 antibody. The mixture was subsequently washed three times with lysis buffer and the RNAs were extracted using Trizol reagent (Invitrogen).

### Immunohistochemistry (IHC) staining

The clinical human RCC samples were fixed in 10% (v/v) formaldehyde in PBS, embedded in paraffin, and cut into 5 μm sections, which were used for IHC staining with specific primary antibody against ASS1. To enhance antigen exposure, the slides were treated with 1 × EDTA at 98 °C for 10 min for antigen retrieval, incubated with endogenous peroxidase blocking solution, and incubated with the primary antibody at 4 °C overnight. After rinsing with Tris-buffered saline, the slides were incubated for 45 min with biotin-conjugated secondary antibody, washed, and then incubated with enzyme conjugate horseradish peroxidase (HRP)-streptavidin. Freshly prepared DAB (Zymed, South San Francisco, CA) was used as a substrate to detect HRP. Finally, the slides were counter-stained with hematoxylin and mounted with aqueous mounting media. Positive cells were calculated as the number of immunopositive cells × 100% divided by the total number of cells/field in 10 random fields at 400× magnification.

### In vivo studies

Thirty-two 6–8 week-old nude mice were purchased from NCI and divided into four groups for injection of OSRC-2 cells transduced with Luciferase and the following constructs: (1) pWPI + pLVTHM; (2) oeAR + pLVTHM; (3) pWPI + oeASS1P3; and (4) oeAR + oeASS1P3. The prepared stable OSRC-2 cells (mixed with Matrigel, 1:1) were injected at 1 × 10^6^ into the subrenal capsule of the mice. The development and metastasis of RCC tumors were monitored using the non-invasive In Vivo Imaging System (IVIS, Spectrum, Caliper Life Sciences, Hopkinton, MA) once a week. The mice were sacrificed after 8–10 weeks. Tumors and metastases were removed for study.

### Statistical analysis

Data were expressed as mean ± SEM from at least three independent experiments with all data points performed in triplicate. Statistical analyses were conducted with SPSS 17.0 (SPSS Inc., Chicago, IL). *P* < 0.05 was considered statistically significant.

## Results

### AR promotes RCC cell proliferation independently of the Von Hippel–Lindau (VHL) status

**Fig. 1:**
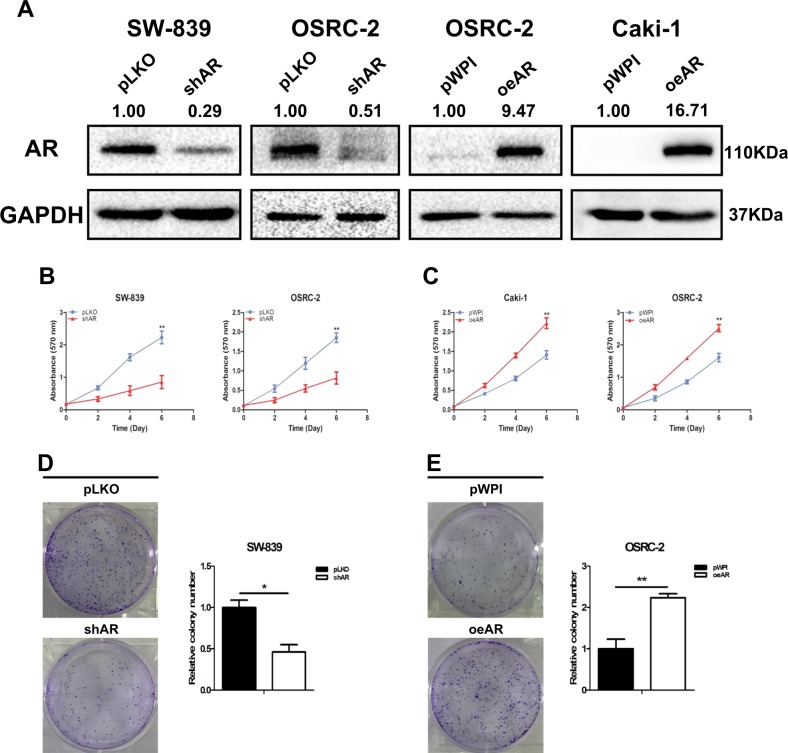
AR promotes RCC cell proliferation independently of the VHL status. **a** Verification of AR overexpression and knockdown by western blot assay in SW-839, OSRC-2, and Caki-1 cells. **b** SW-839 and OSRC-2 cells were transfected with AR-shRNA and negative control separately, and cell growth was measured by MTT assay. **c** Caki-1 and OSRC-2 cells were transfected with functional AR-cDNA and negative control separately, and cell growth was measured by MTT assay. **d**, **e** The cell colony formation in SW-839 and OSRC-2 cells with AR knockdown or overexpression. For **b**–**e**, data are presented as mean ± SEM, *P < 0.05, **P < 0.01 compared to the controls

To study AR’s roles in RCC progression, we first targeted AR by adding either AR-shRNA in VHL-mutant SW-839 and OSRC-2 cells, or functional AR-cDNA in OSRC-2 and VHL-wild-type Caki-1 cells (Fig. 1a). The results of MTT assays revealed that suppressing AR decreased cell proliferation in SW-839 and OSRC-2 cells (Fig. 1b), and increasing AR increased cell proliferation in Caki-1 and OSRC-2 cells (Fig. 1c). In addition, a long-term colony formation assay led to similar results (Fig. 1d, e).

Together, results from Fig. 1a–e suggest that AR can increase RCC cell proliferation independently of the VHL status.

### Mechanism dissection of why AR can increase RCC cell proliferation: by suppressing ASS1 expression

**Fig. 2:**
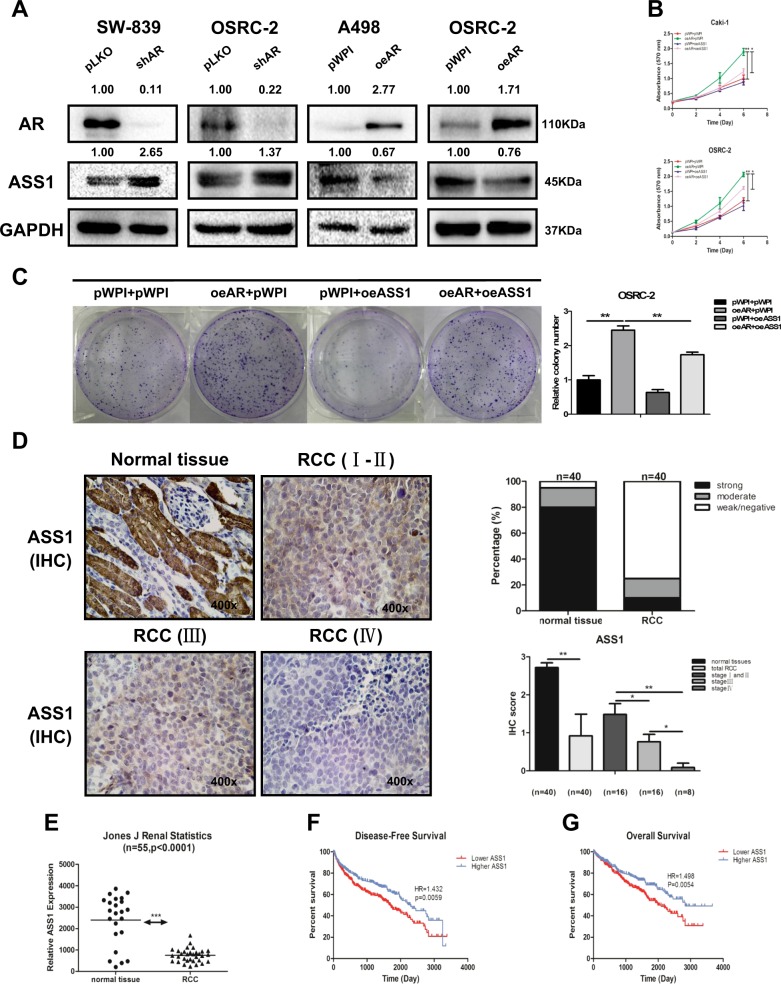
AR could suppress ASS1 expression, and decreased expression of ASS1 is correlated with a worse prognosis in RCC. **a** SW-839 and OSRC-2 cells were transfected with AR-shRNA or negative control. A498 and OSRC-2 cells were transfected with functional AR-cDNA and negative control. The expression of ASS1 was measured by western blot assay. **b**, **c** MTT and colony formation rescue assay reveals that AR-increased cell proliferation could be reversed/blocked after adding ASS1-cDNA in OSRC-2 and Caki-1 cells. **d** Immunohistochemical staining results to detect ASS1 level in 40 paired primary RCC (Stages I-IV) and adjacent normal tissues that were obtained from the Shengjing Hospital of China Medical University, Shenyang, China. Left panels show representative images, while right panels show the quantification using two different methods. Magnification is ×400. **e** Analysis of RCC microarray from NCBI GEO Datasets (GSE15641) shows ASS1 mRNA level in 55 RCC samples. **f**, **g** Disease-free survival curves (**f**) and overall survival curves (**g**) of RCC patients analyzed according to ASS1 expression (data were analyzed from TCGA). For (**b** and **c**), data are presented as mean ± SEM, *P < 0.05, **P < 0.01 compared to the controls

To dissect the mechanisms underlying AR’s promotion of RCC cell proliferation, we focused on the ASS1, as recent studies have indicated that decreased ASS1 activity might lead to an increase in the tumor growth^6,7^. We knocked down AR by adding AR-shRNA and found increased expression of ASS1 in SW-839 and OSRC-2 cells. Similarly, increasing AR by adding AR-cDNA led to decrease in the expression of ASS1 in A498 and OSRC-2 cells (Fig. 2a).

Importantly, results from the interruption approach revealed that AR-increased cell proliferation could be reversed/blocked after adding ASS1-cDNA in OSRC-2 and Caki-1 cells (Fig. 2b). Using colony formation assay, we obtained similar results (Fig. 2c).

Together, results from Fig. 2a–c suggest that AR may function via altering the ASS1 to increase the RCC cell proliferation.

### Human clinical data suggest that decreased expression of ASS1 is correlated with a worse prognosis in RCC

To validate the above in vitro cell line data, we applied IHC staining to examine ASS1 expression in 40 pairs of primary RCC and adjacent normal renal tissues. The results revealed that ASS1 expression was lower in RCC tissues than in adjacent normal renal tissues (Fig. 2d images, left panel), with 75% cases being weak/negative for ASS1 expression and 10% cases with strong positive expression (Fig. 2d, right panel).

Importantly, we found the expression of ASS1 was negatively linked to the tumor stage with less ASS1 expression during tumor progression (Fig. 2d, lower panel). Similar results were also obtained by analysis of Jones J’ microarray raw data in the GEO Datasets (GSE15641) (Fig. 2e). Analysis of TCGA database with more than 600 human RCC samples also revealed that patients with a lower ASS1 expression had a significantly lower disease-free survival (HR = 1.432, *P* = 0.0059) as well as overall survival (HR = 1.498, *P* = 0.0054) than those with a higher ASS1 expression (Fig. 2f, g), respectively.

Together, results from multiple human clinical surveys (Fig. 2d–g) suggest that decreased expression of ASS1 is correlated with a worse prognosis in RCC.

### Mechanism dissection of how AR can suppress the ASS1: by altering the ASS1P3 function

**Fig. 3:**
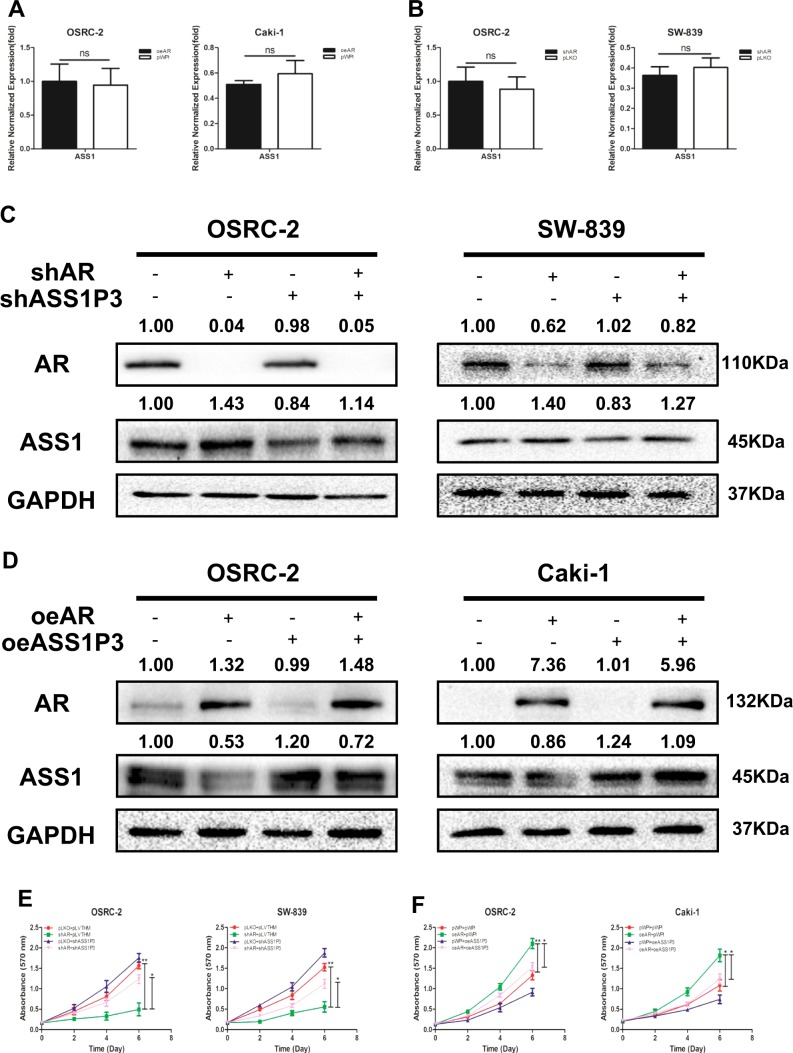

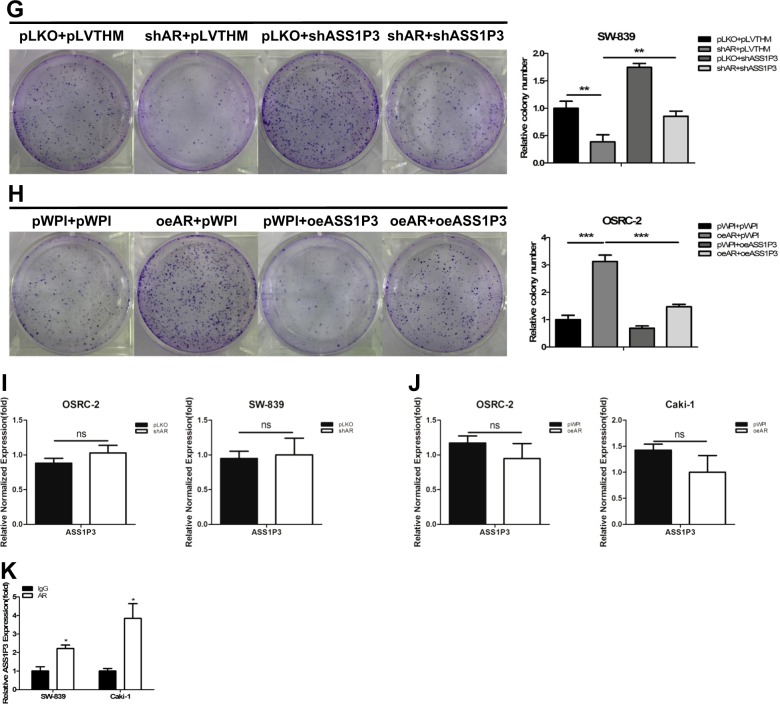
AR could suppress ASS1 to promote RCC proliferation through ASS1P3. **a** Real-time RT-PCR assay was applied to detect the expression of ASS1 in OSRC-2 and Caki-1 cells with overexpressed AR compared with pWPI. **b** Real-time RT-PCR assay was applied to detect the expression of ASS1 in OSRC-2 and SW-839 cells with knocked-down AR compared with pLKO. **c** Western blot assay shows that ASS1P3-shRNA could partially reverse the AR-shRNA-increased ASS1 expression in OSRC-2 and SW-839 cells. **d** Western blot assay shows that ASS1P3 could partially reverse the AR-decreased ASS1 expression in OSRC-2 and Caki-1 cells. **e** MTT assay shows that ASS1P3-shRNA could partially reverse the AR-shRNA-increased ASS1 expression in OSRC-2 and SW-839 cells. **f** MTT assay shows that ASS1P3 could partially reverse the AR-decreased ASS1 expression in OSRC-2 and Caki-1 cells. **g** Colony formation assay shows that ASS1P3-shRNA could partially reverse the AR-shRNA-increased ASS1 expression in SW-839 cells. **h** Colony formation assay shows that ASS1P3 could partially reverse the AR-decreased ASS1 expression in OSRC-2 cells. **i** Real-time RT-PCR assay was applied to detect the expression of ASS1P3 by knocking down AR compared with pLKO in OSRC-2 and SW-839 cells. **j** Real-time RT-PCR assay was applied to detect the expression of ASS1P3 in overexpressed AR compared with pWPI in OSRC-2 and Caki-1 cells. **k** RIP assay was performed to detect the interaction between AR and ASS1P3 after adding AR and IgG to SW-839 and Caki-1 cells, respectively. For A-B, E-F, G-K, data are presented as mean ± SEM, *P < 0.05, **P < 0.01, ns = not significant, compared to the controls

To further explore how AR regulates ASS1 expression, we altered AR and assayed its impact on the expression of ASS1. The qRT-PCR results (Fig. 3a, b) revealed that adding functional AR-cDNA in OSRC-2 and Caki-1 cells (a) or suppressing AR with AR-shRNA in OSRC-2 and SW-839 cells (b) led to minor changes on the ASS1 mRNA expression.

This discrepancy in ASS1 protein and transcript expression suggested an involvement of the ncRNAs, including miRNAs, long noncoding RNAs (lncRNAs), and circular RNAs (circRNAs), that might mediate the AR’s regulation at a post-transcriptional level. Through literature search, we found that ASS1 had 12 pseudogenes that had similar functions with lncRNAs and could regulate their cognate genes as ceRNAs by competing with miRNAs. As seen from the supplementary alignment of ASS1 gene and its pseudogenes (Supplementary Fig. S1A), only ASS1P3 and ASS1P13 share the 3′UTR with the wild-type gene, as well as having a unique segment of sequence that allows specific detection and knockdown. Our initial analysis with knocking down both ASS1P3 and ASS1P13 indicated that only ASS1P3 could result in regulation of ASS1 expression. Therefore ASS1P3 was chosen for further study.

We confirmed that the expression of ASS1P3 was upregulated with pWPI-ASS1P3, and downregulated when knocked down with pLVTHM-shASS1P3 in OSRC-2, SW-839, and Caki-1 cells (Supplementary Fig. S2A).

We found that knocking down AR could increase the expression of ASS1 in both OSRC-2 and SW-839 cells, and this AR-shRNA-increased ASS1 expression could be partially reversed after adding the ASS1P3-shRNA (Fig. 3c). In a reverse manner, we found that ASS1P3 could partially reverse the AR-cDNA-decreased ASS1 expression in OSRC-2 and Caki-1 cells (Fig. 3d).

Using MTT (Fig. 3e, f) and colony formation assays (Fig. 3g, h), we obtained similar results in OSRC-2, SW-839, and Caki-1 cells.

Next we focused on how AR regulates ASS1P3 function. The results of qRT-PCR assay indicated that the RNA level of ASS1P3 changed little compared to the vector controls after manipulation of AR in OSRC-2, SW-839, and Caki-1 cells (Fig. 3i, j). Among many possibilities of how AR could regulate ASS1 through its pseudogene ASS1P3, we tested the possibility of AR directly binding to this RNA. The results of RNA immunoprecipitation (RIP) with a specific AR antibody indicated that AR indeed could directly bind to ASS1P3 (Fig. 3k).

We also detected the expression of AR, ASS1P3, and ASS1 in 40 paired primary RCC tissues and adjacent normal renal tissues. The qRT-PCR results showed that the mRNA level of AR was higher in RCC tissues than in adjacent normal renal tissues (Supplementary Fig. S2B), and the expression levels of both ASS1P3 and ASS1 were lower in RCC tissues than in adjacent normal renal tissues (Supplementary Fig. S2C-D). Furthermore, we found lower AR expression was associated with higher ASS1P3 expression (Supplementary Fig. S2E).

Together, the results from Fig. 3a–k and Supplementary Figs. S1 and S2 indicate that AR can suppress ASS1 to promote RCC cell proliferation likely through binding to ASS1P3.

### Mechanism dissection of how AR/ASS1P3 can suppress ASS1 expression: by altering the miR-34a-5p function

**Fig. 4:**
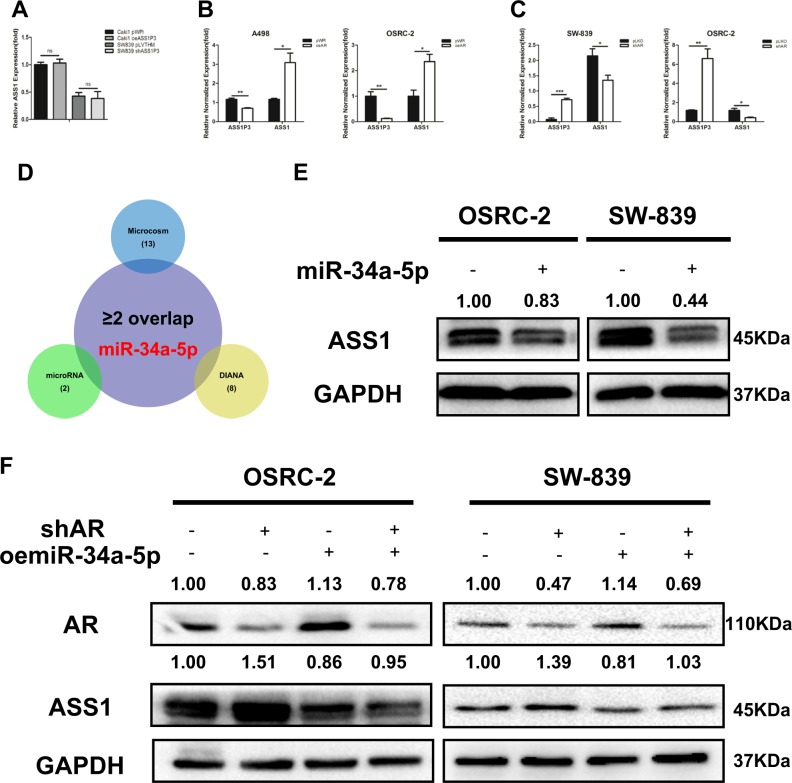
ASS1P3 could regulate ASS1 expression via miR-34a-5p. **a** Real-time RT-PCR assay was applied to detect the expression of ASS1 in Caki-1 cells with overexpressed ASS1P3 and in SW-839 cells with knocked-down ASS1P3. **b** Argonaute2 immunoprecipitation assay was performed after transfecting A498 and OSRC-2 cells with functional AR-cDNA or vector. **c** Argonaute2 immunoprecipitation assay was performed after transfecting SW-839 and OSRC-2 cells with AR-shRNA or vector. **d** Cartoon of screening for miRNA candidates. **e** Western blot assay was applied to detect the expression of ASS1 in OSRC-2 and SW-839 cells after overexpressing miR-34a-5p. **f** Western blot assay showed that oemiR-34a-5p could partially reverse the AR-shRNA-increased ASS1 expression in OSRC-2 and SW-839 cells. For (**a**–**c**), data are presented as mean ± SEM, *P < 0.05, **P < 0.01, ns = not significant, compared to the controls

Next, to examine the molecular mechanism of how AR/ASS1P3 could suppress ASS1 expression in RCC cells, we found that knocking down ASS1P3 decreased ASS1 expression (Fig. 3c, d), and adding ASS1P3 increased ASS1 expression at the protein level, but not at the mRNA level (Fig. 4a), suggesting a potential post-transcriptional regulation that might involve the miRNA(s). We then applied the Argonaute2 immunoprecipitation assay to examine the RNA level in the immunoprecipitates as a gauge of miRNA–mRNA interactions. The results showed that ASS1 mRNA increased and ASS1P3 decreased after adding AR in A498 and OSRC-2 cells, suggesting that AR could bind with ASS1P3 to release some miRNAs, which might then be able to target/decrease the ASS1 expression (Fig. 4b). Consistent with this, knocking down AR in SW-839 and OSRC-2 cells led to an increase of ASS1P3, with a decrease of ASS1 mRNA in the AGO2 immunoprecipitates (Fig. 4c).

A search of the potential miRNA(s) that could bind to both ASS1P3 and ASS1 by bioinformatic analysis (microcosm, microRNA, and DIANA) yielded one candidate, miR-34a-5p, because only miR-34a-5p overlapped in the above three bioinformatic websites (Fig. 4d). We confirmed that the expression of miR-34a-5p was upregulated with pWPI-miR-34a-5p in OSRC-2 and SW-839 cells (Supplementary Fig. S2F). Adding miR-34a-5p in OSRC-2 and SW-839 cells resulted in a significant reduction of ASS1 expression (Fig. 4e). The rescue assay with knocked-down AR led to an increase of ASS1 expression in SW-839 cells, and this AR-shRNA-increased ASS1 expression could be partially reversed after adding the miR-34a-5p (Fig. 4f). Similar results were obtained in SW-839 cells.

Together, the results from Fig. 4a–f suggest that AR/ASS1P3 can suppress ASS1 expression by altering the miR-34a-5p.

### ASS1P3 functions as a miRNA sponge for miR-34a-5p to regulate ASS1 in RCC

**Fig. 5:**
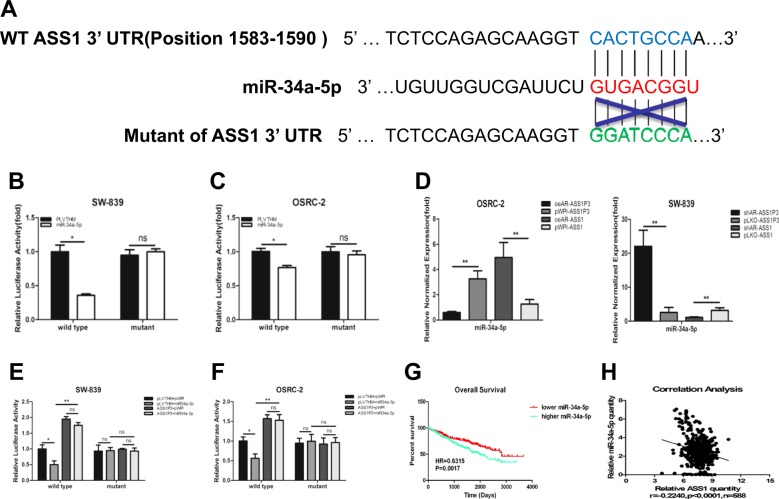
ASS1P3 functions as a miRNA sponge for miR-34a-5p to regulate ASS1 in RCC. **a** Sequence alignment of the ASS1 3′UTR with wild type versus potential mutant miR-34a-5p targeting sites. **b, c** Co-transfection of wild type or mutant seed regions of ASS1 3′UTR constructs with miR-34a-5p in SW-839 (**b**) and OSRC-2 (**c**) cells. The luciferase assay was applied to detect the luciferase activity. **d** Pull-down assay was performed by biotinylated antisense oligo specific for ASS1P3 and ASS1 after transfecting cells with functional AR-cDNA, AR-shRNA, or vector control in OSRC-2 and SW-839 cells. **e, f** Co-transfection of wild type or mutant seed regions of ASS1 3′UTR constructs with miR-34a-5p, ASS1P3, pLVTHM, and pWPI in SW-839 (**e**) and OSRC-2 (**f**) cells for luciferase assay to detect the luciferase activity. **g** Overall survival of RCC patients was analyzed according to miR-34a-5p expression based on the TCGA dataset. **h** Correlation analysis of miR-34a-5p and ASS1 mRNA levels by Pearson correlation coefficient analysis in the TCGA dataset. For **b**–**f**, data are presented as mean ± SEM, *P < 0.05, **P < 0.01, ns = not significant, compared to the controls

To directly implicate miR-34a-5p in regulating ASS1 expression, we generated reporter constructs using the psiCHECK2 vectors carrying the wild-type and mutant miRNA-target sites of 3′UTR of ASS1 (Fig. 5a). The results showed that miR-34a-5p could suppress the luciferase reporter activities of wild-type 3′UTR of ASS1, while no significant differences could be found for the mutant 3′UTR of ASS1 in SW-839 cells (Fig. 5b), suggesting that miR-34a-5p could target the 3′UTR of ASS1 directly to suppress its protein expression. Similar results were obtained when we used OSRC-2 cells (Fig. 5c).

Next, to verify that miR-34a-5p was one of the miRNAs that could directly interact with ASS1P3 and ASS1 and “move” between them, we applied the pull-down assay (Fig. 5d) to detect the level of miR-34a-5p associated with ASS1P3 and ASS1. We used biotinylated antisense oligo to isolate ASS1P3 and ASS1 from the OSRC-2 cells with exogenous AR and SW-839 cells with knocked-down AR, as well as vector controls. The results indicated that ASS1P3 could retain less miR-34a-5p, whereas ASS1 could retain more miR-34a-5p after adding AR in OSRC-2 cells (Fig. 5d). Conversely, knocking down AR in SW-839 cells led to an increase of miR-34a-5p in the ASS1P3 pulldown, but a decrease of miR-34a-5p in the ASS1 pulldown (Fig. 5d).

To test the ability of ASS1P3 to competitively bind to miR-34a-5p, we applied the reporter assay with the psiCHECK2 vector carrying the wild-type and mutant 3′ UTR of ASS1. The luciferase activities decreased in SW-839 cells after adding miR-34a-5p (Fig. 5e), and the inhibitory effect of miR-34a-5p on ASS1 was abolished after ASS1P3 was expressed (Fig. 5e). No significant difference was found for the mutant 3′ UTR of ASS1 in SW-839 (Fig. 5e). Similar results were obtained when OSRC-2 cells were used (Fig. 5f).

Consistent with the role of miR-34a-5p in promoting RCC, we found through TCGA dataset analysis that patients with higher miR-34a-5p expression had a relatively worse OS (HR = 0.6315, *P* = 0.0017) than patients with lower miR-34a-5p expression (Fig. 5g). In addition, a lower ASS1 expression was associated with a higher miR-34a-5p expression in the similar TCGA dataset analysis (Fig. 5h).

Together, the results from Fig. 5a–h indicate that AR can regulate ASS1 by binding to ASS1P3 to differentially regulate miR-34a-5p availability and a pseudogene ASS1P3 function as a miRNA sponge to regulate ASS1 expression through miR-34a-5p in RCC.

### In vivo mice studies confirm that AR promotes the proliferation of RCC via altering the ASS1P3 expression

**Fig. 6:**
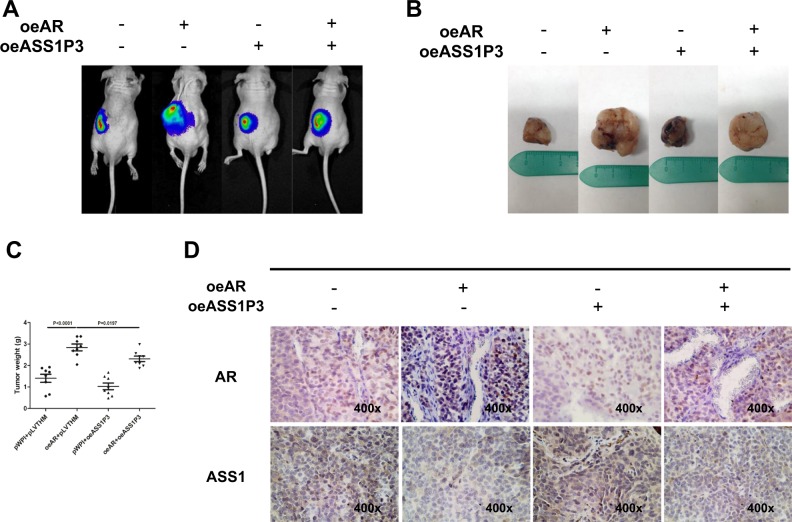
In vivo mice studies confirm that AR promotes proliferation of RCC via ASS1P3. **a** IVIS imaging was used to determine the sizes of the tumors. **b** Gross RCC tumor samples of nude mice after 6 weeks' injection of OSRC-2 cells. **c** Quantification of weights of tumors in each group. **d** Representative images of IHC staining of tumor regions for AR and ASS1

To further confirm all the above in vitro data showing that the pseudogene ASS1P3 might function as a ceRNA mediated by AR to suppress cell proliferation by sponging miR-34a-5p in RCC, we generated four groups of OSRC-2-Luc cells, also transfected with (1) pWPI + pLVTHM, (2) oeAR + pLVTHM, (3) pWPI + oeASS1P3, and (4) oeAR + oeASS1P3, and inoculated these cells (1 × 10^6^) into the left renal capsules of nude mice. We used IVIS to monitor tumor growth every week. After 6 weeks, we found that the mice inoculated with oeAR + pLVTHM cells had a larger tumor size compared to the control group by IVIS (Fig. 6a). We then sacrificed the mice and measured the size and weight of each tumor. The results showed that higher tumor weights were found in the oeAR + pLVTHM group than in the pWPI + pLVTHM group. Adding ASS1P3 led to a reduction in tumor size and weight consistent with its ability to sponge miR-34a-5p, thus enhancing ASS1 expression and activity. Co-expression of AR with pseudogene ASS1P3 could block its suppression likely by inhibiting interaction between miR-34a-5p and ASS1P3, thus releasing the former to suppress ASS1 to promote the tumor growth (Fig. 6b, c). Our IHC staining demonstrated that ASS1 was decreased after enhancing AR expression, while overexpression of ASS1P3 partly blocked/reversed the AR-induced effect (Fig. 6d).

Together, results from the in vivo studies in Fig. 6a–d confirmed our in vitro studies and demonstrate that AR can promote RCC proliferation by modulation of ASS1P3/miR-34a-5p/ASS1 signaling.

## Discussion

Tumor metabolism occupies an important role in tumorigenesis. Some signaling pathways^14,15^ and molecular markers^16^ have been found to be specifically associated with tumor metabolic processes. Panneerselvam et al.^14^ found that the impaired Fanconi Anemia tumor suppressor signaling pathway could predict distinct metabolic signatures of bladder cancer cells. Lu et al.^15^ revealed that acetyl-CoA acetyltransferase 2 could alter cholesterol metabolism and participate in hepatocellular carcinoma formation. Ventrucci M et al.^16^ found that tumor M2-pyruvate kinase could be used as a new metabolic marker for pancreatic cancer. Recently, a key enzyme, ASS1, which can convert citrulline to arginine in the urea cycle, was found to have a frequent low expression in several types of tumors^6,7^. Choy^17^ reported that a low expression of ASS1 in glioblastoma had the potential to be a predictive marker for therapeutic efficacy. Qiu^6^ found that ASS1 was either low or absent in more than 60% breast cancer bio-samples. Consistent with above data, we found that ASS1 expression was lower in RCC tissues than in adjacent normal renal tissues. Furthermore, the expression of ASS1 decreased significantly during RCC progression, which was correlated with a worse prognosis. These findings indicated an important role of ASS1 in the proliferation and progression of RCC.

Increasing evidence has demonstrated that AR may play important roles in tumor proliferation^18–20^, including RCC^21^. The mechanisms underlying AR’s promotion of RCC proliferation and progression are complicated. Here we reported that AR could regulate RCC proliferation by regulating the miRNA availability for ASS1 by repressing the sponge role of its pseudogene ASS1P3, consistent with recent studies showing that pseudogenes could regulate their ancestral genes in disease progression^22–24^. Poliseno et al.^13^ observed that a pseudogene (PTENP1) could regulate coding gene (PTEN) expression and revealed a noncoding function for mRNAs. Yang et al.^24^ found that a FOXO3 pseudogene could regulate FOXO3 activity in the inhibition of tumor growth and angiogenesis. Wang et al.^25^ showed that the pseudogene OCT4-pg4 could regulate OCT4 expression as a miRNA sponge. In our case, we found that a pseudogene (ASS1P3) of ASS1 participated in the AR/ASS1 signal pathway and ASS1P3 expression could partially reverse the AR-decreased ASS1 expression. Increased expression of ASS1P3 could result in reducing cell proliferation, thus a potential therapeutic modality for advanced RCC.

Some studies showed that miR-34a might play important roles as a tumor-suppressor miRNA to target certain critical genes in RCC^26–28^. For example, Yu et al.^26^ found that miR-34a could suppress cell proliferation and metastasis by targeting CD44 in RCC. Yamamura et al.^27^ revealed that miR-34a could target c-Myc transcriptional complex to suppress malignant transformation in RCC. Zhang et al.^28^ found that miR-34a could inhibit cell proliferation by targeting Notch1 in RCC. In extension of these studies, we found that the pseudogene ASS1P3 could serve as a ceRNA to affect the level of its cognate gene expression via miR-34a-5p. A lower expression of ASS1P3 resulted in the suppression of ASS1 via miR-34a-5p, and enhanced cell proliferation. Therefore, miR-34a-5p could act as an oncogene in this scenario, suggesting the role of miR-34a-5p in tumorigenesis is likely context- or stage-dependent.

It is worth mentioning that our cell line studies did not find a relationship in expression between AR and ASS1P3, as it is likely AR undergoes a physical interaction with ASS1P3 to influence its ceRNA activity, while a survey of tumor samples suggested a negative correlation in expression of both genes (Supplementary Fig. S2B-E). It is possible that a long-term impact for such interaction in the context of RCC tumor formation may be different from short-term tissue culture study. Nevertheless, these findings are consistent with the conclusion that AR expression is negatively correlated with ASS1 expression, thus contributing to RCC tumor progression, likely by regulating the ceRNA activity of ASS1 pseudogenes.

In summary, we reported a novel mechanism underlying AR’s promotion of RCC proliferation and progression by AR protein binding of a pseudogene ASS1P3 to suppress the latter’s ceRNA function, thus releasing miR-34a-5p to inhibit ASS1 expression. Such knowledge could provide novel insights as well as potential therapeutic approaches to suppress RCC proliferation and progression.

## Supplementary information

Supplemental Figures

Supplemental figure legends
