# Supplementary material for: Androgen receptor regulates ASS1P3/miR-34a-5p/ASS1 signaling to promote renal cell carcinoma cell growth
Source: Cell Death Dis. 2019 Apr 18;10(5):339. doi: 10.1038/s41419-019-1330-x (PMC6472417; doi:10.1038/s41419-019-1330-x)

Supplementary Fig. S1

Go directly to Alignment

```
Multalin version 5.4.1
Copyright I.N.R.A. France 1989, 1991, 1994, 1996
Published research using this software should cite
Multiple sequence alignment with hierarchical clustering
F. CORPET, 1988, Nucl. Acids Res., 16 (22), 10881-10890
Symbol comparison table: blosum62
Gap weight: 12
Gap length weight: 2
Consensus levels: high=90% low=50%
Consensus symbols:
 ! is anyone of IV
 $ is anyone of LM
 % is anyone of FY
 # is anyone of NDQEBZ

 MSF:   2020    Check:   0          ..
 Name: ass1           Len:  2020  Check: 1310  Weight:  0.79
 Name: ASS1P13        Len:  2020  Check: 4615  Weight:  0.79
 Name: ASS1P3         Len:  2020  Check: 6108  Weight:  0.83
 Name: ASS1P9         Len:  2020  Check: 1637  Weight:  1.03
 Name: ASS1P5         Len:  2020  Check: 8260  Weight:  1.04
 Name: ASS1P12        Len:  2020  Check: 5167  Weight:  1.04
 Name: ASS1P10        Len:  2020  Check: 1405  Weight:  1.04
 Name: ASS1P11        Len:  2020  Check: 1202  Weight:  1.06
 Name: ASS1P7         Len:  2020  Check: 4918  Weight:  1.07
 Name: ASS1P4         Len:  2020  Check: 6945  Weight:  1.04
 Name: ASS1P6         Len:  2020  Check: 5203  Weight:  1.04
 Name: ASS1P2         Len:  2020  Check: 8281  Weight:  1.10
 Name: ASS1P1         Len:  2020  Check:  518  Weight:  1.12
 Name: Consensus      Len:  2020  Check: 6883  Weight:  0.00

//


           1                                                                                                                                         130
      ass1 CTGCTCTGCC GCCTGCCACC GCTGCCCGAG CCCGGTGGGC GGCGGCACTG CGTGCTGGGG AAGCACAGAT TCTTGTGCCC TCTGTGACCT TGAGCCTGCC ACTGACCTCT CTGAGCCCTG CTGGCTGCCT
   ASS1P13
    ASS1P3
    ASS1P9
    ASS1P5
   ASS1P12
   ASS1P10
   ASS1P11
    ASS1P7
    ASS1P4
    ASS1P6
    ASS1P2
    ASS1P1
 Consensus

           131                                                                                                                                       260
      ass1 CCCCAATAGG AATGACAACT CCTACTTCAT GAGGTTGTAG TGGGGGCTCA GGGGCACAGG GAGATCCCGC TGAGGCTCCC AGGGCCCTGC CTGGTGCTTG GCAAGGAGCA GTTCTGATGG GGGATGTGTC
   ASS1P13
    ASS1P3
    ASS1P9
    ASS1P5
   ASS1P12
   ASS1P10
   ASS1P11
    ASS1P7
    ASS1P4
    ASS1P6
    ASS1P2
    ASS1P1
 Consensus

           261                                                                                                                                       390
      ass1 AGCTGCCAGA GGCTCAGAGC CAAGGCCCCT CCCACATCTC CTGCCTGCGT GCATTCCTCA CATCCTGGCC TTGGGCCAAA CTGCTCAGCC TCAGCCGCCT TCCCTCGATG CTGGAGGATC AGGTCCAAGA
   ASS1P13
    ASS1P3                                                                                          CTGC- TCTGTCACCT GCCACCGCTG CC-------C AAGCCTGAGT
    ASS1P9
    ASS1P5
   ASS1P12
   ASS1P10
   ASS1P11
    ASS1P7
    ASS1P4
    ASS1P6
    ASS1P2
    ASS1P1
 Consensus                                                                                          ....  .......... .......... ..       . ..........

           391                                                                                                                                       520
      ass1 GATGCCTCTC CCCGACCAGC CTAGGACTGC AGTCAGCATT CCCAGGCCCC AGTGTGGTCT GTGGGGCTGT GTGCAGAGTG TGCGAGACGC TATGTCCAGC AAAGGCTCCG TGGTTCTGGC CTACAGTGGC
   ASS1P13          T CCTGCTCTGT C---GCCTGC CAT-GGCTGC CCCAG---CC CGAGTGGTTC ATTGCACTGT GGAGTCAGAT TCCG-GACGG TATGTCCAGC AAAGGCTCTA TAGTTCTGGC CTGCAGTGGC
    ASS1P3 GGT-TCACTA CAC------- -----CATGA AGACAGATT- -CCAGATGCC GGCA------ -----ACTG- GTGCTTCCAA TCCTAGACGC TATGTCCAGC AAAGACTCCA TGGTTCTGGC CTACAGTGG-
    ASS1P9                                                                                                  ATGTCCACC AAAGGCTCCG TGGTTCTGGC CTACAGTGGC
    ASS1P5                                                                                                  ATGTCCAGC AAAGGCTCTG TGGTTCTGGC CTACAGTGGC
   ASS1P12                                                                                                  ATGTCCAGC AAAGGCTCAG TGGTTCTGGC CTACAGTGGC
   ASS1P10                                                                                                C TATGTCCAGC AAAGGCTCCA TGGTTCTGGC CTACAGTGGT
   ASS1P11                                                                                                  ATGTCCAGC AAAGGCTCAG TGGTTCTGGC CTACAGTGGC
    ASS1P7                                                                                                     TCCAGC AGAGGCTCCA TGGTTCTGGC CTACAGTGGC
    ASS1P4                                                                                                  GTGTCCAGC AAAGGCTCCA TGGTTCTGGC CCACAGTGGT
    ASS1P6                                                                                                  ATGTCCAGC AAAGGATCCA TGGTTCTGGC CCACAGTGGT
    ASS1P2                                                                                                  ATGTTCAGC AAAGGCTCCA TGGTTCTGGC CTACAGTGCC
    ASS1P1
 Consensus ... ...... .......... .   ...... .......... .......... .......... .......... .......... .......... .atgTcCAgC AaAGgcTCca TgGTTCTGGC CtaCAGTGgc

           521                                                                                                                                       650
      ass1 GGCCTGGACA CCTCGTGCAT CCTCGTGTGG CTGAAGGAAC AAGGCTATGA CGTCATTGCC TATCTGGCCA ACATTGGCCA GAAGGAAGAC TTCGAGGAAG CCAGGAAGAA GGCACTGAAG CTTGGGGCCA
```

```
  ASS1P13  AGCCTGGACA CCTTCTGCAT CCTCGTGTGG CTGAAGGAAC AAGGCTATGA CGTCATTGCC TACCTGGCCA GCATTGGCCA GAAGGAAGAC TTCCAGGAAG CCAGGAAGAA GGCACTGAAG CCTGGGGCCA
   ASS1P3  GGCCTGGACA CCTCCTGCAT CCTCATGTGG CCGAAGGAAC AAGGCTATGA CATAATTGCC TACCTAGCCA ACATTGGCCA GAAGGAAGAC TTCGAGGAAG CCAGGAAGAA GGCACTGAAG CTTGGGGCCA
   ASS1P9  AGCCTGGACA CCTCCTGCAC CCTCCTGTGG CTGAAGGAAC AAGGCTATGA CGTCATTGCC TACCTGGCCA ACATTGGCCA GAAGGAAGAC TTCGAGGAAG CCAGGAAGAA TGCACTGAAG TTTGGGGCTA
   ASS1P5  GGCCTGGACA CCTCCTGCAT CCTCGTGTTA CTGAAGGAAC AAGGCTATGA CATCATTGCC TACGTGGCCA ACACTGGCCA GAAGGAAGAC TTTGAGGAAG CCAGGAAGAA GGCACTGAAG CTTGGGGGCA
  ASS1P12  AGCCCGGACA CCTCTTGCAT CCTCGTGTGG CTGAAGGAAC AAGGCTATGA CATCATTGCC TACCTGGCCA ACACTGGCCA GAAGGAAGAC TTCGAGGAAG CCTGGAAGAA GGCACTGAAG CTTGGGACCA
  ASS1P10  GGCCTGAACA CCTCCTGCAT CCTCGTGTGG CTGAAGGAAC AAGGCTATGA TGTCACTGCC TACCTGGCCA ATACTGGCCA GAAGGAAGAC TTCGAGGAAG CCAGGAAGAA GGCACTGAAG CTTGGAGCCA
  ASS1P11  GGTCTGGACA CCTCGTGTAT CCTCGTGTGG CTGAAGGAAC AAGGCTATGA CGTCACTGCC TACCTGGCCA ACATTGGCCA GAAGGAAGAC TTCGAGGAAG CAAGGAAGAA GGCACCAAAG CTTGGGACCA
   ASS1P7  AGCCTGGACA CCTCCTGCAT CTTTGTGTGG CTGAAGGAAC AAGGCAATGA CATCATTGCC TACCTGGGCA ACATCGGCCA GAAGGAAGAC TTCAAGGAAG CCAGG----- --CACTGAAG CCCGGGGCCA
   ASS1P4  GGCCTGGACA CCTCCTGCAT CCTCATGTGG CTGAAGGAAC AAGGCCATGA TGTCATTGCC TACCTGGCCA AAATCAGCCA GAAGGAAGAC TTCGAGGATG ACAGGAAGAA GGCATGGAAG CTTGGGGCCA
   ASS1P6  GGCCTGGACA CCTCCTGCAT GCTTGTGTGG CTGAAGGAAC AAGGACATGT TGTCATTGCC TACCTGGCCA ACATTGGCCA GAAGGAAGAC TTCGAGAAAG ACAGGAAGAA GGCATGCAAG CTTGGGGCCA
   ASS1P2  GGCCTGGACA CCTCCTGCAT CCTCGTGTGG CTGAAGGAAC AAGGCTATGA CATTATTGCC TACCTGGCCA ATGTTGGCCA GAAGGAAGAC TTCAAGGAAG CCAGGAAGAA GGCACTGAAC CTTGGGTCCA
   ASS1P1                      T CCTCGTGTGG GTGAAGGAAC AAGGCTATGA TGTCATTGCC TACCTGGCCA ACACTGGCCA GAAGGAAGAC TTCGAGGAAG CCAGGAAGAA GGCACTGAAA CTTGGGGCCA
Consensus  gGcCtGgACA CCTccTGcAT CCTcgTGTGG CTGAAGGAAC AAGGCtATGA cgTcAtTGCC TACCTGGCCA AcAttGGCCA GAAGGAAGAC TTCgAGGAAG cCAGGAAGAA gGCActgAAg CtTGGGgCCA

           651                                                                                                                               780
     ass1  AAAAGGTGTT CATTGAGGAT GTCAGCAGGG AGTTTGTGGA GGAGTTCATC TGGCCGGCCA TCCAGTCCAG CGCACTGTAT GAGGACCGCT ACCTCCTGGG CACCTCTCTT GCCAGGCCCT GCATCGCCCG
  ASS1P13  AAAAGATGTT AATTGAGGAT GTCAACAGGG AGTTTGTGGA GGAGTTCATC TGGCCGGCCA TGCAGTCCAG CACACTGTAT GAGGACCGCT ACCTCCTGGG AACCTCTCTC GCCAGGCCCT GCATCACCCA
   ASS1P3  AAAAGGTGTT CATTGAGGAT GCCAGCAGGG AGTTTGTGGA GGAGTTCATC TGGCCGGCCA TCCAGTCCAG GGCACTGTAT GAAGACCACT GCCTCCTGGG CACCTCTCTT GCCAGGCCCT GCATTGCCTG
   ASS1P9  AAAAGGTGTT CATTGAGGAT GTCGGCAGGG AGTTTGTGGA GGAGTTCATC TGGCCGGCCA TCCAGTCCAG TGCACTGTAT GAGGACCGCT ACCTTCTGGG CACCTCTC-- GCCAGGCCCT GCATCGCCTG
   ASS1P5  AAAAGGTGTT CATTGAGGAA GTCAGCAAGG AGTTTGTGAA GGAGTTCATC TGGCTGGCCA TCCAGTCCAG CGCACTGTAT GAGGACCACT ACCTCCTGGG CACCTCTCTC ACCAGGCCCT GCATCGCCCG
  ASS1P12  AAAAGGTGTT CATTGAGGAT GCCAGCAGGG AGTTTGTGGA GGAGTTCATC TGGCCGGCCA TCCAGTCCAG CGCACTGTAT GAGAACCGCT ACCTCCTGGG CACCTCTCTG GCCAGGCCCG GCATCGCCGG
  ASS1P10  AAAAGGTGTT CATTGAGGAT GTCAGCAGGG AGTTTGTGGA GGAGTTCATC TGGGCGGCCA TCCAGTCCAG CGCACTGTAT GAGGACCACT ACCTCCTGGG TACTTCTCTC ACCAGGCCCT GCATCGCCCA
  ASS1P11  AAAAGGTGTT CACTGAGGGT GTCAGCAGGG AGTTTGTGGA GGAGTTCATC TGGCCAGCCA TCCAGTCCAG CTCACTGTAT GAGGACGGCT ACCTCCTGGG CACCTCTCAC ACCAGGCCCT GCACCGCCCG
   ASS1P7  AAAAGGTGTT CACTGAGGAT GTCAGCAGAG AGTTTGTGGA GGAGTTCATC TGGCTGGCCA TCCAGTCCAG TACACTGTAT GAGGACC--- ACCTCCTGGG CAGCTCTCTC CCCAGGCCCT GCATCGCCCA
   ASS1P4  AAAAGGTGTT CATTGATGAT GTCAGCAGGG ACTTTGTGGA GGAGTTCATC TGGCCAGCCA TCCAGTCCAG CATCCTGTAT GAGGACTACT ACCTCCTGGG CACCTCTCTG GCTAGGCCCT GCATCGCCCA
   ASS1P6  AAAAGGTGTT CATTGAGGAA GTCAGCAGGG AGTTTGTGGA GGAGTTCATC TGGCCGGCCA TCCAGTCCAG CGCCCTGTAT GAGGACTACT ACCTCCTGGG CACCTCTCTG GCTAGGCTCT GCATCGCCCA
   ASS1P2  AAAAGGTGTT CATTGAGGAC GTCAGCAAGG AGTTCGTGGA GGAGTTCATC TGGC------ ---------- ----CTGTAT GAGGACTGCT ACCTCCTGGG CCCCTCTCTT CCCAGGCCTT ACATCACCCG
   ASS1P1  AAAAGGTGTT CATTGAGGAT GTCATCAGGG AGTTTGTGGA AGAGTTCATC TGGCCGGCCA TCCAGTCCAG CACACTGTAT GAGGACCGCT ACCTCCTGGG CACCTTTCTC GCCAGGCCCT GCATTGCCAG
Consensus  AAAAGGTGTT CAtTGAGGAt GtCAgCAgGG AGTTTGTGGA GGAGTTCATC TGGCcgGCCA TcCAGTCCAG cgcaCTGTAT GAGGACcgCT ACCTCCTGGG cACCTCTCt. gCcAGGCCCT GCATcgCCcg

           781                                                                                                                               910
     ass1  CAAACAAGTG GAAATCGCCC AGCGGGAGGG GGCCAAGT-A TGTGTCCCAC GGCGCCACAG GAAAGGGGAA CGATCAGGTC CGGTTTGAGC TCAGCTGCTA CTCACTGGCC CCC-C--AGA TAAAGGTCAT
  ASS1P13  CAAACAAGTG GAAGTCGCCC AGCGGGAGGG GGCCAAAT-A TGTGTCCCAC GGCACCACGG GAAAGGGGAA TGATCAGGTC TGGTTTGAGC TCACCTGCTA CTCGCTGGCC CCCAC--AGA TAAAGGTCAT
   ASS1P3  CAAACAAATG GAAATTGTCC AGCAGGAGGG GGCCAAGT-A TGTGTCCCAC GGTGCCACGG GAAAGGGGAC CGATCAGTTC TGATTTGAAC TCACCTGCTA CTCACTGGCC CCCCCCCAGA TAAAGGTCAT
   ASS1P9  CAAACAAATG GAAATCACCC AGTGGAAGGG GGCCAAGT-A TATGTCCCAC AGCACCACGG GAAATGGGAA CAATCAGGTC CGGTTTGAGC TCATTTGCTA CTCACTAGCC CCCC---AGA TAAAGGTCAT
   ASS1P5  AAAACAAGTG GAAATCGCCC AGCAGGAGAG GGCCAAGT-A AGTGTCCCAC AGCGCCACAG GAAAGGGAAA TGATCAGGTC CAGTTTGAGC TAAACTGCTA CTCTCTGGCC CCCCC--AGA TAAAGGTCAT
  ASS1P12  CAAACAAGTG GAAATCGCCA AGCAGGAGGA GGCCAAGT-A TGTGTTCCAC GGCACCACGG GAAAAGGAAA TGATCAGGTC CGGTTTGAGC TCACCTGCTA TTTGCTGGCC CCCC---AGA TAAAGGTCAC
  ASS1P10  CAAAAAAGTG GAAACCGTCC AGCGGGTGGG GGCCAAGT-G TGTGTCCCAC AGCCCCACAA GAAAGGGGAA CGATCAGGTC CAGTTTGAGC TCATCTGCTA CTCGCTGGCC CCCC---AGA TGAAGGTCCC
  ASS1P11  CAAACAAGTG GAAATCCCCC AGCGGGAGGG GGCCAAGTTA TGTGTGTCAT GGC------- ---------- ---TCAGGTC TGGTTTGAGC TCACCTGCTA CTCACTGGCC CCCC---AGA TAAAGGTCAT
   ASS1P7  CAAACAAGTA GAAATCACCC AGCAGGAGGG GGCCAAGT-G TGTGTCCCAT GGCACCACGG GAAAGGGCAA TGATCAGGCC CGGTTC-AGC TCACCTGCTA CTCGCTGGCC CCCCC--AGA TAAAGGTCAT
   ASS1P4  CAAACAAGTG GAAATCACCC AGCAGGAGGG GACCAAGT-A TGTATCCTAC GGCGTCATGG GAAAGGGGAA CGATCATGTC CTGTTTGAGC TCACCTGCT- --CACTGGCC CTC----AGA TTAAGGTCAT
   ASS1P6  CAAACAAGTG GAAATTACCC AGCAGGAGGG GGCCAAGT-A CGTATCCTAT GGCATCATGG GAAAGGAGAA CGATCAGGTC CAGTTTGAGC TCACCTGCT- --CACTGGCC CCC----AGA TTAAGGTCAT
   ASS1P2  CAAACAAGTG GAAATTGCTC AGTGGGAGGG GGCAAAGT-A TGTGTCCCAC AGTGCCATGG GAAAGGGGAA CGATCAGGTC TGGTTTGAGC TCGCCTGCTA CTCGCTGGCC CCCC---AGA TTAAGGTCAT
   ASS1P1  CAAACAAGTG GAAATCGCCC AGCAGGAGGG G--------- -GTGTCCCGC GGCGCCACCG GTAAGGGGAA T-ATCAGGTC CGGTTTGAGC TCACCTGGTA CTCGCTGGTC CCCC---AGA TAAAGGGCAC
Consensus  CAAACAAgTG GAAATcgcCC AGcaGGAGGG GgCcAAgT a tGTgTcCcAc gGc.cCAcgg GaAAgGggAa cgATCAGGTC cgGTTTGAGC TCAcCTGCTa ctC.CTGGCC CCCc.  AGA TaAAGGTCAt

           911                                                                                                                              1040
     ass1  TG-----CTC CCTGGAGGAT GCCTGAATTC TACAACCGGT TCAAGGGCCG CAATGACCTG ATGGAG---T ACGCAAA-GC AACACGGGAT TCCCATCCCG GTCACTCCCA AGAACCCGTG GAGCATGGAT
  ASS1P13  TG-----CTC CCTGAAGTAT GCCCGAATTC TACAACCGGT TCAAGGGCCA CAGTGACCTG ACGGAA---T ATGCAAA-GC AACGCGGGAT TCCCACCCCG GTCACTCCCA AGAACCCGTG GAGCATGGAC
   ASS1P3  TG-----CTC CCTGGAGGAT GCCCGAGTTC TACAACTGGT TCAAGGCCTT CAATGACCTG ATGGAAGAAT ACACAAAAGC AACACGGGAT TCCCATCCTG GTCACTTCCA AGAAGCCATG GAGCATGGAA
   ASS1P9  TG-----CTC CCTGGAGGAT GCCCAAATTC TACAACCAGC TCAAGGGCCG CAATGACCTG ATGGAA---T ATGCAAA-GC AACACAGGAT TCCCATCCCG GTCACACCCA AGAACTCGTG GAGCATGGAC
   ASS1P5  TG-----CTC CCTGGAGGAT GCCCAAGTTC TACAACAGGT TCAAGGTCCG AAATGACCTT ATGGAA---C ACACAAA-GC AACACGGGAT TCCCATCCCA GTCACTCCCA AGAACCTGTG GAACATGGAC
  ASS1P12  CG-----CTC CCTGGAGGAT GCCCGAGTTC TACAACCGGT TCAAGGGCTG CAATGACCTG ATGAAA---T AGGCAAA-GC AACATCGGGT TCCCATTCCA GTCACTCCCC AGAGCCCGTG AAGCATAGAC
  ASS1P10  TGGAGGGCTC CCTGGAGGAT GCCCGAGTTC TACAACTGGT TCAAGGGCCG CAGTGACCTG ATGGAA---T ATGCAAA-GC AACACGGGAT TCCCATCCTG GTCACTCCCA AGAACCTGTG GAGCATCGAC
  ASS1P11  TG-----CTC CCTGGAGCAT GCCCGAATTC TACAACCACT TCAAGGGCTG CAGTGACCTG ACGGAA---T ACACAAA-GC AACACGGGAT TCCCATCCCA GTCACTCCCA AGAACCCGTG GAACATGGAC
   ASS1P7  TG-----CTC CCTGGTGCAT GTTCGAGTTC TACAATCGGA TCAAGTGCCT CAACGATCTG ATGGAA---T ATGCAAA-GC CACACGGGAT TCCCATCCCA GTCACTCCCA AGAACCCGTG GAGCATGGAT
   ASS1P4  TG-----CTT CCTGGAGGAT GCCCGAGTTT TACAGCTGGT TCAAGGGCCT TAATGATCTC ATGGAA---T ACGCAAA-GC AACATCGGAT TCCCATCCCA GTCACTCTCA AGAACCCGTG GAGCATAGAC
   ASS1P6  TG-----CTC CCTGGAGGAT GCCCGAGTTC TACTACTGGC TCAATGGCTG CAATGATCTG ATGGAA---T ACACAAA-GC AACATCGGAT TCCCATCCCA GTCACTCTCA AGAACCCGTG GATCATGGAC
   ASS1P2  TG-----CTC CCGGGAGGAT CCCTGAGTTC TACAACCAGT CCAAGGGCCG CAGTGATCTG ATGGAA---T ATGCAGA-GA AACATGGGAT TCCCATCCCA GTCACTCTGA AGCACCCATG GAACATGGAC
   ASS1P1  TG-----CTC CCTGGAGGAT GCCCGAGTTC TACAACCGGT TCAAGGGCCG TAATGACCTG ATGGAA---T ACGCAAA-GC AGCACGGGAT TCCTATCCCG GTCACTCCCA AGAACCCGTG GAACATGGAC
Consensus  TG     CTC CCTGGAGgAT GCCcgAgTTC TACAACcgGt TCAAGGgCcg cAaTGAcCTg AtGGAA   T AcgCAAA GC AACAcgGGAT TCCCATCCca GTCACTCcCA AGAACCcgTG GAgCATgGAc

           1041                                                                                                                             1170
     ass1  GAGAACCTCA TGCACATCAG CTACGAGGCT GGAATCCTGG AGAACCCC-A AGAACCA--A GCGCCTCCAG GTCTCTACAC GAAGACCCAG GACCCAGCCA AAGCCCCCAA CACCCCTGAC ATTCTCGAGA
  ASS1P13  GAGAACCTCA TGCACATCAG CTGCGAGGCT GGAATCCTGG AGAATCCC-G AGAACCA--A GCGCCTCCAG CTCTCTACAC GAAGACCCAG GACCCGGCCA AAACCCCCAA CACCCCTGAC ATTCTCGAGA
   ASS1P3  CAGAACCTCA TGCACATCAG CTGTGAGGCT GGAATCCTGG AGAACCCC-A AGAACTA--A GTGCCTCCAG GTCTCTACAC GAAGACCCAG GACCTGGCCA AAGCCCCCAA CACCCCTGAT ATTCTCGAG-
   ASS1P9  GAGAACCTCA TGCATATCAG CTACGAGTCT GGAATCCTGG AGAACCCT-A AGAACCA--A GCACCTCCAG GTCTCTACAC GAAGACC-AG GACCCGGCCA AAGCCCCCAA CACCCCTGAC ATTCTCGAGA
   ASS1P5  GAGAACCTCA TGCAGATCAG CAATGAGGCT GGAATCTTGG AGAACCCT-A AGAACCA--A GCATTTCCAG GTCTCTCCAC GAAGACCCAG GACCCGGCCA AAGCCCCCAG AACCCCTGAC ATTCTCGAGA
  ASS1P12  GAGAACCTCA TGCACATCAG CTATGAGGCT GGAATCCTGG A--ACCCC-A AGAACCA--A GTGCCTCCAG GTCTCTACAT GAAGACCCAG GACCCAGCCA AAGCCCTCAA CACCCCTGAC ATTCTCGAGA
  ASS1P10  AAGAACCTCA TGCACATCAG CTACAAGGCT GGAATCCTGG AGAACCCC-A AGAACCA--A GTGCCTCCAG GTCTCTACAC AAAGATCCAG GACCCAGCCA AAGCCCCCAA CACCCCTGAC ATTCTCAAGA
  ASS1P11  GAGAACCTCA TGCACATCAG CTACGAAGCT GGAATCCTAG AGGACCCCCA AGAACCA--A GCGCCTCCAG GTCTCTACCC GAAGACCCAG GAC-TGGCCA AAGCCCCCAA CACCCCTGAC ATTCTCGAGA
   ASS1P7  GAGAACCTCA TGCACATCAG CTATGAGGCT GGAATCCTGG AGAGCCCC-A AGAACCA--A GCGCCTCCAG GTTGCTGCAC GAAGACCCAG GACCCAGCCA AAGCCC-CAA CACCCCTGAC ATTCTCAAGA
   ASS1P4  GAGAACCTCA TGCATATCAG CTACGAGGCT GGAATTCTGG AGAAACCC-A AGTACCATAA GCACCTCCAG GTCTCTACAT GAAGACTAAG GACCTCACCA AAGCCCCCAA CACCACTGAC ATTCTCGAGA
   ASS1P6  GAGAACCTCA TGTGTATCAG CTATGAGGCT GGAATCCTGG AGAAACAC-A AGTACCATAA GCACCTCCAG GTCTCTACAC AAAGACTAAG GACCTGACCA AAGCCCCCAA CACCCCTGAC ATTCTCTAGA
   ASS1P2  GAGAACCTCA TGCACATCAG CCACGAGGCT GGAATCTTGG AGAACCCC-A AGAACCA--A CCACCTTCAG GTCTCTACAT GAAGATTCAG GACCTGGCCA AAGCCCCCAA CACCCCCAAC ATTTTCAAGA
   ASS1P1  GAGAACCTCA TGCACATCAG CTACGAGGCT GGAATCCTGG AGAACCCC-A AGAACCA--A GTGCCTCCAG GTCTCTACAC GAAGACCTAG GAACCGGTCA AAGCCCCCAA CACCCCTGAC ATCCTCGAGA
Consensus  gAGAACCTCA TGCAcATCAG CtacGAGGCT GGAATCcTGG AGaAcCCc A AGaACCA..A GcgCCTCCAG GTCTCTaCAc gAAGAcccAG GACCcggCCA AAGCCCcCAA CACCCCTGAC ATTCTCgAGA

           1171                                                                                                                             1300
     ass1  TCGAGTTCAA AAAAGGGGTC CCTGTGAAGG TGACCAACGT CAAGGATGGC ACCACCCACC AGACCTCCTT GGAGC----- -------TCT TCATGTACCT GAACGAAGTC GCGGGCAAGC ATGGCGTGGG
  ASS1P13  TCGAGTTCAA AAAAGGGGTC CCCGTGAAGG TGACCAACGT CAAGGATAGT GCCACCCACC AGACCTCCTT GGAGC----- -------TCT TCATGTACCT GAACGAAGTC GCGGGCAAGC ACAGCGTGGG
   ASS1P3  -----TTCCA AAACGGGGTC CCTGTGAAGG TGACCAACGT CAAGGATGGC ACCACCCACC AGACCTCCTT GGTACCTTGG TACCTCCTTT TCATGTACCT AAACGAAGTC ACGGGCAAGC AGGGTGTAG-
   ASS1P9  TCGAGTTCAA AAAAGGAGTC CCCGTGAAGG TGACCAACGT CAAGGATGGC ACCACCCACC AGACCTCATT GGAGC----- -------TCT TCATGTACCT GAACGAAGTC GTGGGCAAGC AGGGTGTGGG
   ASS1P5  TCGAGTTCAA ATAAGGAGTC CCCATGAAAG TGACCAACGT CAAGGATGGC ACCACCCACC AGACCTCCTT GGAGC----- -------TCT TCCTGTACCT GAACGAAGAC GTGGGCAAGT ACAGCTTGGG
  ASS1P12  TCAAGTTCAA AAAAGGGGTC CCCGTGAAGG TGACCAACGT CAAGGATGGC ACCACCCACC AGACCTCCTT GGAGC----- -------TTT TCGTGTACCT GAATGAAGTT GCAGGCAAGC ACGGCGTGGG
  ASS1P10  TCCAATTCAA AAAAGGAGTC CCCGTGAGGG TGACCAGCGT CAAGGATGGC ACCACCCACC AGACCTCCTT GGAGC----- -------TCT TCATGTACCT GAACGAAGTT GCAGGCAAGC ACGGTGTGGG
  ASS1P11  TCGAGTTCAA AAAAGGGGTC TTCTTGAAGG TTACCAACGT CAAGGATGGC ACCACCCACC AGACCTCCTT GGCGC----- -------TCT TCATGTACCT GAATGAAGTC ACGGGAAAGC ACTGCGTGGG
   ASS1P7  TCGAGTTCAC AAAAGGGGTC CCAGTGAAGG TGACCAACAT CAAGGATGGC ACCATCCACC AGACCTCCTT GGAGC----- -------TCT TCGTGTACCT GAACGAAGTC GCGGGCAAGC ACCGTGTTGG
   ASS1P4  TTGAGTCCAA AAAAGGGGTC CCTGTGAAGG TGACCAACGT CAAGGATGGC GCCACCCACC AGACCTCCTA GGAGC----- -------TCT TCATGTACCT GAACCAAGCT GCAAGCAAGC ATGGCCTGGG
   ASS1P6  TTGAGTTCAA AAAAGAGGTC CCCGTGAAGG TGACCAACGT CAAGGATGGC GCCACCCACC AGACCTCCTA GGAGC----- -------TCT GCGTGTACCT GAACGAAGCT GCGAGCAAGC ATGGCCTGGG
   ASS1P2  CTGAGT--AA AAAAGGGGTC CCTGTGAAGG TGACCAGCAT CAAGGATGGC ACCACCCACC AGACCTCCTT GGTGC----- -------TCT TCATGTACCT GAATGAAGTC GCAGGCAAGC ACAGCGTGGG
   ASS1P1  TCGAGTT-AA AAAAGGGGTC CCCGTGAAGG TGACCAACGT CAACGATGGC TCCACCCACC CGACCTCCTT GGAGT----- -------TCT TCATGTACCT GAACGAAGTC GCGGACAAAC ACGGTGTGGG
Consensus  tcgAgTtcAA AAAAGGgGTC CCcgTGAAGG TGACCAaCgT CAAGGATGGC aCCACCCACC AGACCTCCTt GGaGC             TcT TCaTGTACCT GAAcGAAGtc gcggGCAAGC AcgGcgTgGG

           1301                                                                                                                             1430
     ass1  CCGTATTGAC ATCGTGGAGA ACCGCTTCAT TGGAATGAAG TCCCGAGGTA TCTACGAGAC CCCAGCAGGC ACCATCCTTT ACCATGCTCA TTTAGACATC GAGGCCTTCA CCATGGACCG GGAAGTGCGC
  ASS1P13  CAATATTGCC ATCGTGGAGA ACCGTTTCAT TGGAACGAAG TCCCGAGGTA TCTACCGGGC CCCAGCAGGC ACCATCCTTT GCTACGCTCA TTTAGACATC GAGGCCTTCA CCATGTACCG GGAAGTGCAC
   ASS1P3  CCATACTGAC ATCGTGGAGA ACCGCTTCAT TGGAATGAAG TCCCAAGGTA TCTAGGATAC CCCAGCAGGC ACCATCCTTT ACCACGCTCA TTTAGACATC AAGGCCTTCA CCATGGACCT GGAAGTGCGC
   ASS1P9  CTGTATTGAC ATCATGGAGA ACCGCTTCAT TGGAATGAAG TCCCGAGGGA TCTACCAGGC CCCAGCAAGC ACCATCCTTT ACCTCGCTCA TTTAGACACC AAGGCCTTCA CCATGGGCCA GGAAGTGCGC
   ASS1P5  CCGTATTGAC ATCAAGGAGA ACCACTTCAC TGGAATGAAG TCCCCAGGTA TCTATGAGAC CCCAGCAGAC ACCATCGTTT ACCATGCTCA TTTAGACATC AGGGCCTTCA CCATGGACCG GGAAGTATGC
  ASS1P12  CCGTATTGAC ATCGTGGAGA ACCGCTTCAT TGGAATGAAG TCCTGAGGTA TCTGCGAGGC CCCAGCAGGG ACCATCCTTC ACCACACTCA TTTAGACATC AAGGCCTTCA CCATGTACCG AGAAGTGCGC
  ASS1P10  CT-TATTGCC ATCGTGGAGA ACATCTTCAC CAGA-CAAAG GCCCAAGGTA TCTACCAGAC CCCAGTAGGC ACCATCCTTT ACCATGCTCA TTTAGACATC AAGGTCTTCA CCACAGACCG GGAAGTGCAC
  ASS1P11  [illegible]
```

```
  ASS1P11  [illegible]
  ASS1P7   CTCTATTGAC ATCATGGATA ACCGCTTCAT TGGAATGAGG TCCCGAGGTA TCTACGAGAC CCCAGCAGAC ACCATCCTTT ACCACGCTCA TTTAGACATT GAGGCCTTCA CCATGGACCA GTAAGTGTGC
  ASS1P4   CCATATTGAC ATCGTGGAGA ACCATTTCAT TACAATGAAG TCCTGAGGCA TCTATGAGAC CCCAGCAGGC ACCATCCTTT ACCATGCTCA TTCACACATC CAGGCCTTCA CCATGGATCG GGAAGTGCGC
  ASS1P6   CCATATTGAC ATCGTGGAGA ACCATTTCAT TGCAATGAAG TCCTGAGGCA TCTACAAGAC CCTAGCTGGC ATCATCCTTT ACTATGCTCA TTTACACATC GAGGCCTTCA CCATGAATCA GGAAGAGTGC
  ASS1P2   CCATATTGAC ATCGTGGAGA ACCGCTTCAT TGAAATGAAG TCCTGAGGTA TCTGCAAGAC CCCAGCAGGC ACCATCCTTT ACCACCCTCA TTTAGACATT GAGGGCTTTG CCATGGAACA GGAAGTGCGC
  ASS1P1   CTGTACTGAC ATCGTGGAGA ACCGCTTCAC TGGAATGAAG TCCCGAGGTA TCTATGAGAC CCCAGCAGGC ACCATCCTTT ACCACACTCA TTTAGACATC AAGGCCTTCA CCATGGACTG GGAGGTGTGC
Consensus  Cc.TAtTGaC ATCgTGGAGA ACcgcTTCAt TggAAtGAAG TCCcgAGGtA TCTacgAGaC CCCAGCAGgC ACCATCCTTT ACcAcgCTCA TTTAgACATc .AGGcCTTCA CCATGgAccg GGAAGTGcgC

           1431                                                                                                                               1560
     ass1  AAAATCAAAC AAGGCCTGGG CTTGAAATTT GCTGAGCTGG TGTATACCGG TTTCTGGC-- ------ACAG CCCTGAGTGT GAATTTGTCC GCCACTGCAT CGCCAAGTCC CAGGAGCGAG TGGAAGGGAA
  ASS1P13  AAAATCAAAC AATGCCGGGG CTTGAAATTT GCTGAGCTGG TGTATACTGG TTTCTGGCTT TCCGGCACAG CCCTGAGTGT GAATTTGTCC GCCACTGCAT CGCCGAGTCC CAGGAGTGAG TGGAAGGGAA
  ASS1P3   AAAATCAAAC AAGGCCTGGG CTTGGAATTT GCTGAGCTGG TGTATACCGG TTTCTGGC-- ------ACAG ACCAGAGTGT GAATTTGTCC GCCACTGCAT TGCCAAGTTC CAGGAGCGAG TAGAAGGGAA
  ASS1P9   AAAATCAAAC AAGGCCTGGG CTTGAAATTT GTTGAGCTGG TGGATACCGG TTTCTGGT-- ------ACAG CCCTGAGTGT GAATTTGTCT GCCACTGCAT CGCCAAGTCC CAGGAGCAAG TGGAAGGGAA
  ASS1P5   AAAATCAAAT AAGGCCTGGG CTTGAAATTT GCTGAGCTGG TGTATACCGG TTTCTGGC-- ------ACAG CCTTGAGTGT AAATTTGTCC ACCACTGCAT TGCCAAGTCC CAGGAGTGAG TGGAAGGGAA
  ASS1P12  AAAATCAAAC AAGGCCTGAG CTTGAAATTT GCTGAGCTGG TGTATACCGG TTTCTGGC-- ------ATAG CCCTGAGTGT GAATTTGTCC GCCACTGTAA TGCCAAGTCC CAGGAATGAG TGGAAGGGAA
  ASS1P10  AAAACCAATC AAGGCCTGGG CTTGAAATTT GCTGAGCTGA TATACACCAG TTTCTGAC-- ------ATAG CCCTGAGTGT GAATTTGTCC GCCCCTGCAT CGCCAAGTCC CAGGAGCCGG CAGAAGGGAA
  ASS1P11  AAAATCAAAC AAGGCCTGGG CTTGAAATGT GCTGAGCTGG TGTATACTGG TTTCTGGC-- ------ACAG CCCTGAGTGT GAATTTGTCC GCCACTCCAT CGCCAAGTCC CAGGAGCGAG TGGAAAGGAA
  ASS1P7   AAAATCAAAC AAGGCCTGGA CTTGAAATTG GCTGAGCTGG TGTACACCGG TTTCTGGC-- ------ACAG CCCTGAATGT GAACGTGTCC GCCACTGCAT CACCAAGTCC CAGGAATGAG TGGAAGGGAA
  ASS1P4   AAAATCAAAT AAGGCCTGGG CTTGAAATTT GCTGAGCTGC TGTACACCGG TTTCTGGC-- ------ACAG CCCTGAGTGT GAATTTGTCC GCCACTACAT CGTCAAGTCC CAGGAGCTAG TGGAAGGGAA
  ASS1P6   AAAATCAAAC AAGGCCTGGG CGTGAAATTT GCTGAGCTGG TGTACACCAG TTTCTGGC-- ------ACAG CCCTGAATGT GAATTTGTCC ACCACTGCAT CACCAAGTCC CAGGAGTGAG TGGAAGGGAA
  ASS1P2   AAAATCAAAC AAGGCCTGGG CTTGAAATTT GCTGAGTTGG TGTACACCGG TTTCTGGC-- ------ACAA CCCTCAGTGT GACTTTGCCC ACCACTGCAT TGCCAAGTCC CAGGACCGAG TGGAAGGGAA
  ASS1P1   AAAATCAAAC AAGGCCTGGG CTTGAAATTT GCTGAGCTGG TGTACACCGC TTTCTGGC-- ------ATAG CCCTGAGTGT GAATTTGTCC GCCACTGCAT CGCCAAGTCC TAGGGGCAAG TGGGAGGGAA
Consensus  AAAATCAAAc AAGGCCTGGG CTTGAAATTT GCTGAGCTGg TGTAtACcgG TTTCTGGC         AcAG CCCTGAgTGT GAATTTGTCC gCCACTgCAT cgCCAAGTCC CAGGAgcgAG TgGAAGGGAA

           1561                                                                                                                               1690
     ass1  AGTGCAGGTG TCCGTCCTCA AGGGCCAGGT GTACATCCTC GGCCGGGAGT -CCCCACTGT CTCTCTACAA TGAGGAGCTG GTGAGCATGA ACGTGCAGGG TGATTATGAG CCAA----CT GATGCCACCG
  ASS1P13  AGTGCAGGTG TCTGTCCTTA AAGGCCAGGT GTACATCCTT GGCTGGGAGT GCCCCACTGT CT----ACAA CGAGGAGCCG GTGAGCATGA ACGTGCAGGG TGATTATGAG CCAA----TT GATGCCACCG
  ASS1P3   AGTGCAGGTG TCCGTCCTCA AGGGCCAGGT GTACATCCTC AGCTGGGAGT -ACGCACTGT CTCTCTACAG CCAGTTGCTG GTGAGCATGA AAGTGCAGGA TGATTATGAG CCAA----TT GATGCCATCA
  ASS1P9   AGTGCAGGTG TCCGTCCTCG AGGGCCAGGT GTACATCCTT GGCCAGGAGT -CCCCACTGT CTCTCTACAA GGAGGAGCCG GTGAGCATGA ACGTGCAGGG TGATTATCAG TCAA----TT GATGCCACCG
  ASS1P5   AGTGCAGGTG TCCGTCTTCA AGGGCCTGGT GTACATCCTA GGCCAGGAGT --CCCTCTGT TTATCTACAA CGAGGAGCTG GTGAGCATGA ACGTGCAGGT TGATTATGAG CCAA----TC GATGCCACCA
  ASS1P12  AGTGCAGGTG TCTGTCCTCA AGGGCCAGGT GTATATCCTC GGTCGGGAGT -CCCCACTGT CTCTCTACAA CGAGGAGCTG GTGAGCATGA ACGTGCAGGG GGATTATGAG CCGA----TT GATGCCA-CG
  ASS1P10  AGTGCAGGTG CCGGTCCTCA AGGGCCAGGT GTACATCCTT GGCTGGGAGT -CCCCACTGT CTCTCTACAA TGAGGAGCTG ATGAGCGTGA ACGTGCAGGG TGATTATGAG CCGA----TT GATGACACCG
  ASS1P11  AGTACAGGTG TCCCTCTTCA AGGACCAGGT GTACATCCTC GGCTGGGAGT -CCCCACGGT CTCTCTACAA CGAGGAGCTG GTGAGCATGA ACGTGCATGG TGATTATGAG CCAA----TT GATGCCACCG
  ASS1P7   AGTGCAGGTG TCTGTCCTCA AGGGCCAGGT GTACATCCTC CGCTAGGACT -CCCCACTGT CTCTCTACAA TGAGGAGCTG GTGAACATGA ATGTGCAGGA CCATTAGGAG CCAA----TC GATGCCACCG
  ASS1P4   AGTGCAGGTG TCCGTCTTCA AGGGCCAGGT ATACATCCTC GGACAGGAGT -CCCCACTGC CTCTCTACAA CGAGGAGCTG GTGAGCATGA GTGTGCAGGG CGATTAGGAG CCAG----TT GATGCCACCG
  ASS1P6   AGTGCAGGTG CCTGTCTTCA AGAGCCAGGT GGACATACTC AGACAGGAGT -CCCCACTGT CTCTCTAAAA TGAGGAGCTG GTGAGCATGA ATGTGCAGGG CGATTAGGAG CCAA----TT AATGCCACCA
  ASS1P2   AGTGCAGGTA TCCATCTTCA AGGGCCAGGT GTACATCCTC TGCCAGGAGC -CCCCACTGT CTCTCTACAG TGGAGAGCAG GTGAGCATGA ACGTGGAGGG CAATGATGAG CCAGCCAGTC GGTGACACCA
  ASS1P1   AGCGCAGGTG TCCGTCCTCA AGGGCCGGGT GTACATCCTT GGCTGGGAGT -CCCCACTCT CTCCCTACAA CGAGGAGCCG GTGAGCATGA ACGTGTAGGG TGATTATGAG CCAA----TT GATGCCACCG
Consensus  AGTGCAGGTG tCcgTCcTCA AGGGCCaGGT GTACATCCTc gGccgGGAGT  cCCCACTGT CTctCTACAa cGAGGAGCtG GTGAGCATGA AcGTGcAGGg tgATTAtGAG CCaa     Tt GATGcCAcCg

           1691                                                                                                                               1820
     ass1  GGTTCATCAA CATCAATTCC CTCAGGCTGA AGGAATA--- ---TCATCGT CTCCAGAGCA AGGTCACTGC CAAATAGACC CGTGTACAAT GAGGAGCTGG GGCCTCCTCA ATTTGCAGAT CCCCCAAGTA
  ASS1P13  GGTTCATCAA CATCAATTCC CTCAGGCTGA AGGAATA--- ---TCATCGT CTCCAGAGCA AGGTCACTGC AAAATAGACC CCCGTACAAG GAGGAGCTGG GGCCTCCTCA ATTTGCAGAT CGCCCAAGTA
  ASS1P3   GGTTCATCAA CATCAATTCC CTCACGCTGA GGGAATA--- ---TCATCGT CTCCAGAGCA AGGTCACTGC CAAATAGACC CCTGTACAAT GAGGAGTTGG GGTCTCCTCA ATTTGCAGAT CCCCCAAGTA
  ASS1P9   GGTTCATCAA CATCAATTCC CTCAGGCTGA AGGAATA--- ---TCATCGT CTCCAGAGCA AGGTCACTGC CAAATAG
  ASS1P5   GTTTCATCAA CATCAATTCC CTCAGGCTGA AGGAATA--- ---TCATCAT CTCCAGAGCA AGGTCACTGC CAAATAG
  ASS1P12  GGTTCATCAA CGTCGATTCC CTCAGGCTGA AGGAATG--- ---TCATC-- -TCCAGAGCA AGGTCACTGC C
  ASS1P10  GGTTCATCAA CATCAACTCC CTCAGACTGA AGGAATA--- ---CCATTGT CTCCAGAGTA AGTTCACTGC CAGATAG
  ASS1P11  GTTTCATCAA CATCAATTCC CTCAGGCTGA AGGAATA--- ---TCATCAT CTCCAGAGCA AGGTCACTGC CAA
  ASS1P7   GGTTCATCAA CATCAATTCC CTCAGGCTGA AGGAATA--- ---TCACCGT GTCCAGAACA AGGTCACTGC CAAATAGA
  ASS1P4   GGTTCATCAC CATCAGTTCC CTTAGGCTGA AGGAATA--- ---TCATCAT CTCCAGAGCA ACGTCACTGC CAAA
  ASS1P6   GGTTCATCAA CATCAATTCC ATCAGGCTGA AGGAATA--- ---TCATTGT CTCTAAAGCA AGCTCCCTGC CAAA
  ASS1P2   GTCTCATCAA CATCAATTCC CTCAGGATGA AGGAATA--- ---TCATCAT CTCCAGAGCA AGGTCACTGC CAAATAGAC
  ASS1P1   GGTTCATCAA CATCAATTCC CTCAGGCTGA AGGAATAGGA ATATCATCGT CTGCAGAGCA AGGTCACTGC CAAATAGA
Consensus  GgTTCATCAA CATCAATTCC CTCAGGCTGA AGGAATA          TCATcgT cTCCAGAGCA AGgTCACTGC CAaatag... .......... .......... .......... .......... ..........

           1821                                                                                                                               1950
     ass1  CAGGCGCTAA TTGTTGTGAT AATTTGTAAT TGTGACTTGT TCTCCCCGGC TGGCAGCGTA GTGGGGCTGC CAGGCCCCAG CTTTGTTCCC TGGTCCCCCT GAAGCCTGCA AACGTTGTCA TCGAAGGGAA
  ASS1P13  AAGGCGCTAA TTGTTGTGAT AATTTGTACT TGTGACTAGT TCTCCCCGGC TGGCAGCATA GTGGGGCTGC CAGGCCCCAG CTTTGTTCCC TGGTCCCCTT GAAGCCTGCA AACGTCGTCA TCGAAGGGAA
  ASS1P3   CAGGCGCTAA TTGTTGTGAT AATTTGTAAC TGTGACTTGC TCTCCCCGGC TGGCAGCATA GTGGGGCTGC CAGGCTCCAG CTTTGTTCCC TGGTCCCCCT GAAACCTGCA AACGTCATCA TTGAAGGGAA
  ASS1P9
  ASS1P5
  ASS1P12
  ASS1P10
  ASS1P11
  ASS1P7
  ASS1P4
  ASS1P6
  ASS1P2
  ASS1P1
Consensus  .......... .......... .......... .......... .......... .......... .......... .......... .......... .......... .......... .......... ..........

           1951                                                                  2020
     ass1  GGGTGGGGGG CAGCTGCGGT GGGGAGCTAT AAAAATGACA ATTAAAAGAG ACACTAGTCT TTTATTTCTA
  ASS1P13  GGATGGGGGG CAGCTGCGGT GGAGAGCTGT AAAA-TGACA A----AAGAT ACACTAGTC
  ASS1P3   GGG-GCGGGG CAGATGGAGT GGGGAGCTAT AAAA-TTACA ATTAAAAG
  ASS1P9
  ASS1P5
  ASS1P12
  ASS1P10
  ASS1P11
  ASS1P7
  ASS1P4
  ASS1P6
  ASS1P2
  ASS1P1
Consensus  .......... .......... .......... .... ..... .......... .........
```

Supplementary Fig. S2

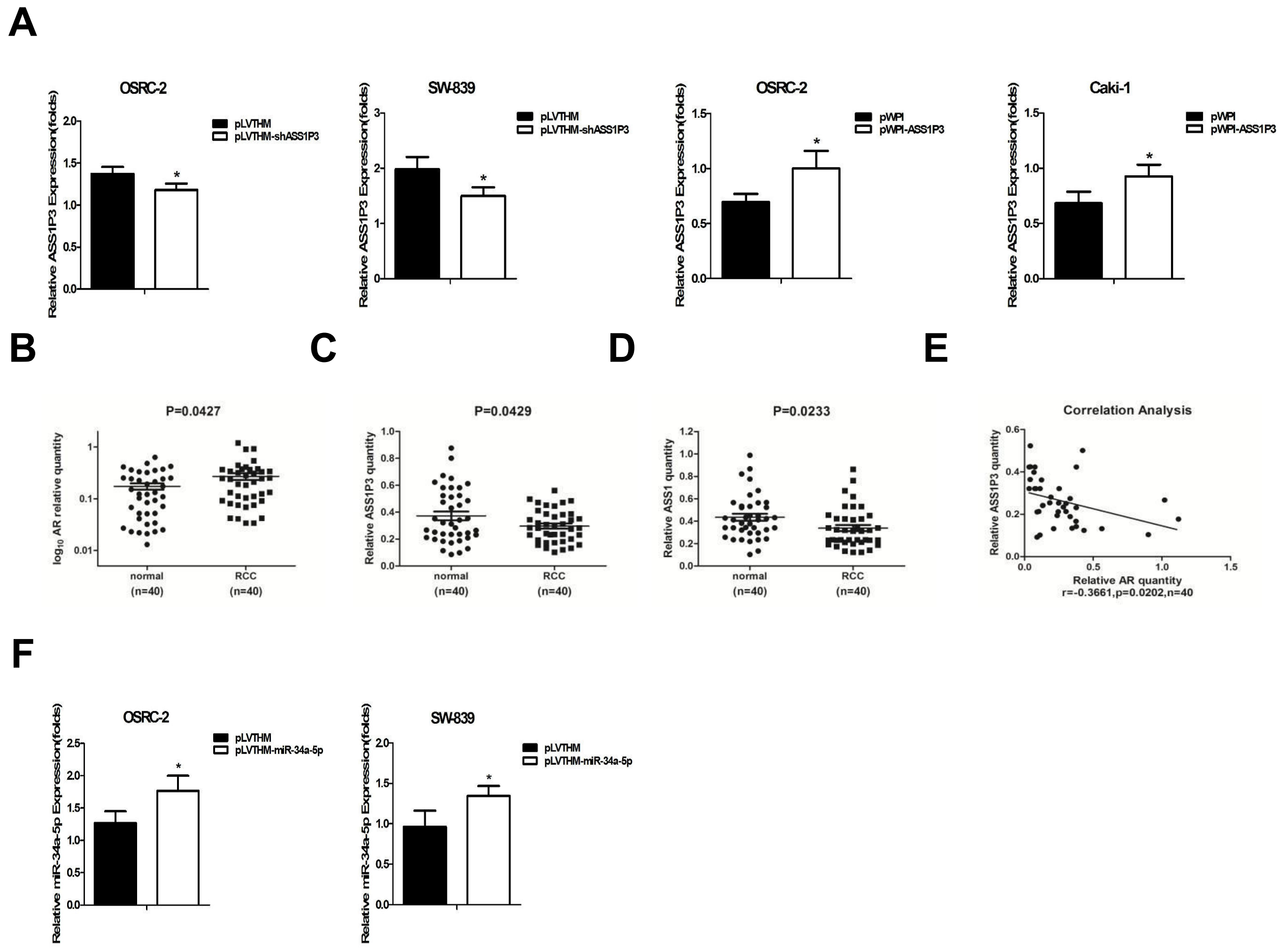

Supplement: Supplementary file 1 — Supplemental Figures [file 41419_2019_1330_MOESM1_ESM.pdf]
