# Supplementary material for: Androgen receptor regulates ASS1P3/miR-34a-5p/ASS1 signaling to promote renal cell carcinoma cell growth
Source: Cell Death Dis. 2019 Apr 18;10(5):339. doi: 10.1038/s41419-019-1330-x (PMC6472417; doi:10.1038/s41419-019-1330-x)
Supplement: Supplementary file 2 — Supplemental figure legends [file 41419_2019_1330_MOESM2_ESM.docx]

**Supplementary Figure Legends**

**Supplementary Figure S1.**

(**A**) Sequence alignment of ASS1 gene and its pseudogenes.

**Supplementary Figure S2.**

(**A**) Verification of ASS1P3 knock down and over-expression by real-time RT-PCR assay in OSRC-2, SW-839 and Caki-1 cells. (**B-D**) AR (**B**), ASS1P3 (**C**) and ASS1 (**D**) expression in 40 human paired primary RCC and adjacent normal tissues. (**E**) Correlation analysis of ASS1P3 and AR mRNA level was performed by Pearson correlation coefficient. (**F**) Verification of miR-34a-5p over-expression by real-time RT-PCR assay in OSRC-2 and SW-839 cells. For A and F, data are presented as Mean ± SEM, *P<0.05 compared to the controls.
